# Complete Mitochondrial Genome of the Fungal Biocontrol Agent *Trichoderma atroviride*: Genomic Features, Comparative Analysis and Insight Into the Mitochondrial Evolution in *Trichoderma*

**DOI:** 10.3389/fmicb.2020.00785

**Published:** 2020-04-28

**Authors:** Yunyoung Kwak

**Affiliations:** ^1^Écologie, Systématique et Évolution, CNRS, Université Paris Sud (Paris XI), Université Paris Saclay, AgroParisTech, Orsay, France; ^2^School of Applied Biosciences, Kyungpook National University, Daegu, South Korea; ^3^Institute for Quality and Safety Assessment of Agricultural Products, Kyungpook National University, Daegu, South Korea

**Keywords:** mitochondrial genome, *Trichoderma*, hypocreales, genomic features, intron, comparative genomics, evolutionary genomics

## Abstract

The improvement of biopesticides for use in the agriculture industry requires an understanding of the biological- and ecological principles underlying their behavior in natural environments. The nuclear genomes of members of the genus *Trichoderma*, which are representative fungal biocontrol agents, have been actively studied in relation to the unique characteristics of these species as effective producers of CAZymes/secondary metabolites and biopesticides, but their mitochondrial genomes have received much less attention. In this study, the mitochondrial genome of *Trichoderma atroviride* (Hypocreales, Sordariomycetes), which targets wood-decaying fungal pathogens and has the ability to degrade chemical fungicides, was assembled *de novo*. A 32,758 bp circular DNA molecule was revealed with specific features, such as a few more protein CDS and *trn* genes, two homing endonucleases (LAGLIDADG-/GIY-YIG-type), and even a putative overlapping tRNA gene, on a closer phylogenetic relationship with *T. gamsii* among hypocrealean fungi. Particularly, introns were observed with several footprints likely to be evolutionarily associated with the intron dynamics of the *Trichoderma* mitochondrial genomes. This study is the first to report the complete *de novo* mitochondrial genome of *T. atroviride*, while comparative analyses of *Trichoderma* mitochondrial genomes were also conducted from the perspective of mitochondrial evolution for the first time.

## Introduction

Mitochondria, double membrane-bound organelles widely considered to have originated from an early alpha-proteobacterium endosymbiont, have various roles for the cell viability in eukaryotic organisms. As well as their primary function as a source of energy production [i.e., adenosine triphosphate (ATP) production] via oxidative phosphorylation (Saraste, 1999), mitochondria are known to be involved in ion homeostasis, intermediary metabolism and cell apoptosis (Burger et al., 2003). Mitochondria harbor their own genetic materials, and mitochondrial DNAs have generally been reported to occur in the form of circular-mapping molecules typically consisting of a single chromosome (i.e., mitochondrial genome or mitogenome) (Burger et al., 2003). The mitochondrial genome is mainly composed of two major gene sets, coding for (1) key components involved in energy production and (2) mitochondrial translation (e.g., transfer RNA), but its genomic structures (e.g., gene content and gene density) varies significantly between eukaryotic lineages (Unseld et al., 1997; Salinas-Giege et al., 2015).

Fungal mitochondrial genomes usually contain 14 conserved core-genes that encode functional proteins involved in oxidative phosphorylation and electron transport, two genes for small and large subunits of ribosomal RNA (rRNAs), and a varying number of genes for transfer RNAs (tRNAs) (Gray et al., 1999; Saraste, 1999). However, the overall size of the mitochondrial genome differs within fungal families, even for closely related species. Approximately 661 fungal mitochondrial genomes are currently available in the GenBank database on NCBI (organelle genome database as of, February 2020: 511 genomes from *Ascomycetes* species, 118 from *Basidiomycetes* species, and 32 from other fungi). Except for a mitochondrial genome of the edible mushroom *Morchella importuna* that reported newly in the size of 272 kb (GenBank accession no. MK527108) (Liu et al., 2020), the largest fungal mitochondrial genome was reported in the phytopathogenic fungus *Rhizoctonia solani* AG3, being ≈235 kb in size with harboring 127 coding sequences (CDS) (GenBank accession no. NC_021436) (Losada et al., 2014), but the mitochondrial genome of *R. solani* AG1 from the same species is only 162 kb in size, with 59 CDS (GenBank accession no. HF546977) (Wibberg et al., 2013). Beyond gene content, other genomic features were characterized in highly variable across fungal mitochondrial genomes, such as gene order, intergenic regions, tRNAs distribution, mobile elements, and intron numbers and size (Aguileta et al., 2014).

Mitochondrial genomes, typically uniparentally inherited (Birky, 1995), are considered as ideal tools for research into eukaryotic evolution due to their (1) relatively small sizes, (2) high copy numbers, (3) irreversible gene loss and limited recombination among mitochondrial genomes, and (4) high mutation rates leading to accelerated evolution independently of nuclear genomes (Burger et al., 2003). However, these unique characteristics contribute differently to the divergent evolutions of the mitochondrial genomes within major eukaryotic taxa. For instance, length heteroplasmy (i.e., the coexistence of different sized mitochondrial genomes) is much more common in animals than in plants and fungi, whereas plant mitochondrial genomes display extensive more variations in genome size and more frequency of recombination than those of animals (Barr et al., 2005). Although fungal mitochondrial genomes have been less studied than those of animals or plants, it seems that they have more complex features of evolutionary patterns. Fungi are phylogenetically positioned close to animals, but fungal mitochondrial genomes show signals of recombination, similar to those of plants (Basse, 2010; Aguileta et al., 2014; Wu and Hao, 2019). Interestingly, however, the introns of fungal mitochondrial genomes have mostly been classified as group I introns, whereas plants generally possess group II introns in their mitochondrial genomes (Lang et al., 2007).

Hypocrealean fungi (Hypocreales, Ascomycota) are both ecologically and economically important. Because of their diverse range of lifestyles as plant-pathogens, plant-saprobes, plant-endophytes, mycoparasites, or pathogens of insects and nematodes (Bushley et al., 2013), hypocrealean fungi act as significant regulators of insects and phytopathogenic fungi in natural environments, and are used as attractive alternatives to chemical pesticides in agriculture (i.e., biocontrol agents) (Rossman et al., 1999). In particular, species of the genus *Trichoderma* (teleomorph *Hypocrea*), which are frequently isolated from free soil, soil litters, dead wood, and the rhizosphere, have been widely used as representative fungal biocontrol agents due to their specific biological abilities, such as (1) growing on the mature sporocarps of phytopathogenic fungi as mycoparasites, (2) utilizing dead fungal biomass as saprotrophs, and (3) inducing plant defense responses by various secondary metabolites, thus increasing plant immunity and the production of cell-wall degrading enzymes protecting plants against phytopathogens (Druzhinina et al., 2011, 2012; Mukherjee et al., 2013).

The improvement of biopesticides for use in the agriculture industry requires an understanding of the biological- and ecological principles underlying their behavior in natural environments. The rapid advance in sequencing technologies has led to an increase in the genome sequencing and analysis of *Trichoderma* species, with the resulting chromosomal genetic information actively analyzed in terms of the molecular regulation- and evolutionary mechanisms of the genus (Martinez et al., 2008; Kubicek et al., 2011, 2019; Mukherjee et al., 2013; Druzhinina et al., 2018). However, to date, only four *Trichoderma* mitochondrial genome sequences are available in the GenBank database on NCBI, i.e., those of *T. reesei* QM9414 (GenBank accession no. AF447590), *T. asperellum* B05 (GenBank accession no. NC_037075), *T. hamatum* (GenBank accession no. MF287973), and *T. gamsii* KUC1747 (GenBank accession no. KU687109). Except for a brief description of the mitochondrial genome of *T. reesei* QM9414 in a study of mitochondrial activity with glucose metabolites (Chambergo et al., 2002), the characterization and comprehensive analysis of these *Trichoderma* mitochondrial genomes have not yet been conducted.

The fungal strain *T. atroviride* ATCC 26799 (=IFO/NBRC 30543; formerly known as *T. harzianum* (Ban et al., 2010); Hypocreales, Sordariomycetes, Ascomycota) is a biocontrol agent that is used to target wood-decaying fungal pathogens and that can degrade chemical fungicides containing chlorinated phenol compounds (Cserjesi, 1967; Highley and Ricard, 1988). Based on previous studies that have reported more rapid mitochondrial evolution under harsh natural conditions (Baker et al., 2017) and mitochondrial activities in response to fungicide effect (Mosbach et al., 2017), the mitochondrial genomic information of the fungus *T. atroviride* ATCC 26799 (=IFO/NBRC 30543) can be useful resources to understand its particular features as both a mycoparasite and a degrader of chemical compounds *in situ*. In addition, the knowledge of this fungal mitochondrial genome will allow expanding our understanding of fungal mitochondrial genomes and its dynamic evolutions in hypocrealean fungi.

## Materials and Methods

### Fungal Strain

The *Trichoderma atroviride* (formerly known as *T. harzianum*; Ban et al., 2010) strain was obtained from the American Type Culture Collection {ATCC, Manassas, VA, United States; accession number ATCC 26799 [=IFO 30543, Institute for Fermentation (IFO, Osaka, Japan)], [=NBRC 30543 NITE Biological Resource Center (NBRC, Chiba, Japan)] (Cserjesi, 1967). The strain ATCC 26799 has presented under the previous name *T. harzianum* in the ATCC collection till now. However, in this study, this fungus was designated as *T. atroviride* ATCC 26799 than *T. harzianum* ATCC 26799 based on a recent report about *Trichoderma* re-identification under the NBRC collection (Ban et al., 2010).

### DNA Extraction and Genome Sequencing

The fungal strain was cultured on Difco^TM^ potato dextrose medium (Difco Laboratories Inc., Detroit, MI, United States) at 25°C for 5 days. Total genomic DNA was extracted from the mycelia using a Plant/Fungi DNA Isolation Kit (Sigma-Aldrich Co Ltd., St. Louis, MO, United States) according to the manufacturer’s instructions and followed by further purification steps using Phenol-Chloroform (v:v, 1:1; Sigma-Aldrich Co Ltd., St. Louis, MO, United States) (Sambrook and Russell, 2006). The quality and quantity of the extracted DNA were assessed using DNA agarose gel electrophoresis (Sambrook, 2001) and Qubit assay using a Qubit^TM^ 3.0 Fluorometer (Thermo Fisher Scientific Inc., Waltham, MA, United States), respectively.

The extracted genomic DNA of *T. atroviride* ATCC 26799 was used to construct a 20-kb insert SMRTbell^®^ DNA library on a BluePippin^TM^ size-selection system (Pacific Biosciences, Menlo Park, CA, United States). It was then sequenced on a Single Molecule Real-Time (SMRT) sequencing platform using a PacBio RS-II DNA sequencer with P6 polymerase-C4 sequencing chemistry (Pacific Biosciences, Menlo Park, CA, United States) (Eid et al., 2009) at the Génome Québec Innovation Centre, McGill University (Canada).

### *De novo* Assembly and Annotation of the Mitochondrial Genome

A total of 1,214,216,973 raw read bases were generated with 450,876 reads from the long-read SMRT sequencing on three SMRT cells. After removing low-quality reads, the remaining raw reads (those with a quality higher than 0.85) were used for *de novo* mitochondrial genome assembly using the Hierarchical Genome-Assembly Process (HGAP) (Chin et al., 2013) in the SMRT^TM^ pipeline (Pacific Biosciences, Menlo Park, CA, United States). The accuracy and circularity of the *de novo* mitochondrial genome assembly were then verified using the P-mapping (BLASR) module (Chaisson and Tesler, 2012) in the SMRT^TM^ pipeline (Pacific Biosciences, Menlo Park, CA, United States) and Gepard (Krumsiek et al., 2007) to generate dotplots, respectively.

The *T. atroviride* ATCC 26799 mitochondrial genome was annotated on webservers for Mitos^1^ (Bernt et al., 2013) and Mfannot^2^ (Valach et al., 2014), under the Genetic Code 4 (the Mold, Protozoan, and Coelenterate Mitochondrial Code). These initial annotations were then modified by the NCBI Open Reading Frame (ORF) Finder (Coordinators, 2018) and BLAST searches of the NCBI non-redundant (nr) database with default parameters (O’Leary et al., 2016). Transfer RNA genes (tRNAs) were annotated by combining the results from the RNAweasel webserver^3^ (Lang et al., 2007) and tRNAScan-SE (v.2.0) (Chan and Lowe, 2019), under the Genetic Code 4. Finally, annotations relating to gene boundaries (particularly at exon-intron junctions) were curated manually to avoid frame-shift errors in the open reading frames (ORFs). In addition, the intergenic region between the genes in the complete *de novo* mitochondrial genome was estimated manually. All annotations for the gene components of the *T. atroviride* ATCC 26799 mitochondrial genome were illustrated in circular plots using Circos (Krzywinski et al., 2009).

### Genomic Analysis of the Mitochondrial Genome

The base composition and codon usage of the *T. atroviride* ATCC 26799 mitochondrial genome were analyzed using MEGA (v.10.0) (Kumar et al., 2018) and the EMBOSS package (Rice et al., 2000). The secondary structure of tRNAs was predicted using tRNAScan-SE (v.2.0) (Chan and Lowe, 2019), and the asymmetric bias of nucleotide composition was assessed according to the following formulas (Perna and Kocher, 1995): AT-skew = (A−T)/(A+T) and GC-skew = (G−C)/(G+C).

The non-synonymous substitution rate (Ka), synonymous substitution rate (Ks), and Ka/Ks ratio for 13 core protein-coding genes (*atp6*, *atp8*, *cob*, *cox1*, *cox2*, *cox3*, *nad1*, *nad2*, *nad3*, *nad4*, *nad4L*, *nad5*, and *nad6*; the *atp9* gene was excluded because it was absent from the mitochondrial genome of *T. gamsii* KUC1747) in the *Trichoderma* mitochondrial genomes were calculated on the pairwise alignments of the target genes between *Trichoderm* species using DnaSP (v.6.12.03) (Rozas et al., 2017), and the obtained values were plotted using ggplot2 (Wickham, 2016) in R (v.3.4.0) (R Core Team, 2017).

To identify repetitive elements in the complete *de novo* mitochondrial genome, interspersed repeats were analyzed using BLAST (Boratyn et al., 2013) searches of the complete mitochondrial genome against itself (self-comparisons: *E*-values < 10^–10^), and tandem repeats were investigated using Tandem Repeats Finder (v.4.0.9) (Benson, 1999) with the default parameters. Lastly, comparative analyses of the gene orders [*rns* and the 14 core genes (*atp6*, *atp8*, *atp9*, *cob*, *cox1*, *cox2*, *cox3*, *nad1*, *nad2*, *nad3*, *nad4*, *nad4L*, *nad5*, and *nad6*)] and the genomic synteny among the *Trichoderma* mitochondrial genomes were carried out in Mauve (v.2.4.0) (Darling et al., 2010) and the results were plotted using Circos (Krzywinski et al., 2009).

### Phylogenetic Analysis

To place the *T. atroviride* ATCC 26799 mitochondrial genome in a phylogenetic tree, complete mitochondrial genomes of 38 *Hypocreales* species (Sordariomycetes, Ascomycota) were downloaded from the GenBank database on NCBI^4^ (Sayers et al., 2019). In addition, one *Sordariales* genome (strain *Neurospora crassa* OR74A; Sordariomycetes, Ascomycota) was downloaded to be used as an outgroup. In the obtained nucleotide datasets, the gene encoding ATP synthase F0 subunit 9 (*atp9*) was absent from the mitochondrial genome of *T. gamsii* KUC1747 (GenBank accession no. KU687109). Therefore, except for *atp9*, all of the sequences from the 13 core genes (*atp6*, *atp8*, *cob*, *cox1*, *cox2*, *cox3*, *nad1*, *nad2*, *nad3*, *nad4*, *nad4L*, *nad5*, and *nad6*) were used for phylogenetic analysis. The nucleotide sequences of the 13 core genes were individually aligned using MAFFT (v.7.429) with an automatic algorithm (parameter: mafft – auto) (Katoh and Standley, 2013), and multiple sequence alignments were concatenated in SequenceMatrix (v.1.8) (Vaidya et al., 2011).

The best-fit evolutionary model for the concatenated sequences was determined using Jmodeltest (v.2.1.10) with the Akaike Information Criterion (AIC) and Bayesian Information Criterion (BIC) (Darriba et al., 2012), the GTR+Gamma+I substitution model was selected as the best fit from all of the different criteria. Based on the established model parameters, phylogenetic relationships were reconstructed using maximum likelihood (ML) (Cavalli-Sforza and Edwards, 1967) and Bayesian inference (BI) (Rannala and Yang, 1996; Nascimento et al., 2017). ML analysis was conducted in RAxML (v.8.2) with 1,000 bootstrap replicates (Stamatakis, 2014), and the bootstrap (BS) values were displayed on the nodes of the constructed phylogenetic tree. BI analysis was carried out in MrBayes (v.3.2.6) (Ronquist et al., 2012), which was run simultaneously with the Markov Chains Monte Carlo (MCMC) algorithm (three heated chains and one cold chain, with a heating coefficient of 0.1) for 2 × 10^6^ generations, and trees were sampled every 400 generations. The first 25% of the sampled trees (by 5 × 10^5^ generations) were discarded as burn-in, and the remaining trees were used to construct the consensus tree with values of Bayesian Posterior Probabilities (BPP). Lastly, the stationarity of BI analysis was considered by calculating the average standard deviation for split frequencies (<0.01).

### Analysis of Mitochondrial Introns

Intron loci of mitochondrial genome were investigated on the RNAweasel webserver^3^ (Lang et al., 2007) under the Genetic Code 4, and intronic open reading frames were identified using the NCBI Open Reading Frame (ORF) Finder (Coordinators, 2018) and BLAST searches of the NCBI non-redundant (nr) database with default parameters (O’Leary et al., 2016).

The evolution of mitochondrial introns was assessed on the Bayesian consensus tree of *Trichoderma* mitochondrial genomes as follow: The nucleotide sequences from exons of 13 core genes (*atp6*, *atp8*, *cob*, *cox1*, *cox2*, *cox3*, *nad1*, *nad2*, *nad3*, *nad4*, *nad4L*, *nad5*, and *nad6*; the *atp9* gene was excluded because it was absent from the mitochondrial genome of *T. gamsii* KUC1747) were individually aligned using MAFFT (v.7.429) with an automatic algorithm (parameter: mafft – auto) (Katoh and Standley, 2013), and multiple sequence alignments were concatenated in SequenceMatrix (v.1.8) (Vaidya et al., 2011). To these concatenated sequences, the best-fit evolutionary model was determined using Jmodeltest (v.2.1.10) (Darriba et al., 2012), and BI analysis was carried out in MrBayes (v.3.2.6) (Ronquist et al., 2012) with the same conditions for the *Hypocreales* phylogeny under the best fit for GTR+Gamma substitution model. The presence/absence patterns of intron loci were mapped on the obtained BI phylogeny according to the state of presence (scored as 1) or absence (scored as 0), and the gain/loss events of introns were traced using the parsimony method in Mesquite (v.3.6) (Maddison and Maddison, 2018). In the reconstructed ancestral states, the presence of a given intron character (*cob*-*i*1, *cox1*-*i*, and *cox3*-*i*) was colored as black on the nodes, and the mitochondrial genome of *Hirsutella thompsonii* ARSEF 9457 (Hypocreales, Ophiocordycipitaceae) was used as an outgroup.

## Results

### Genomic Features of the *T. atroviride* ATCC 26799 Mitochondrial Genome

**FIGURE 1:**
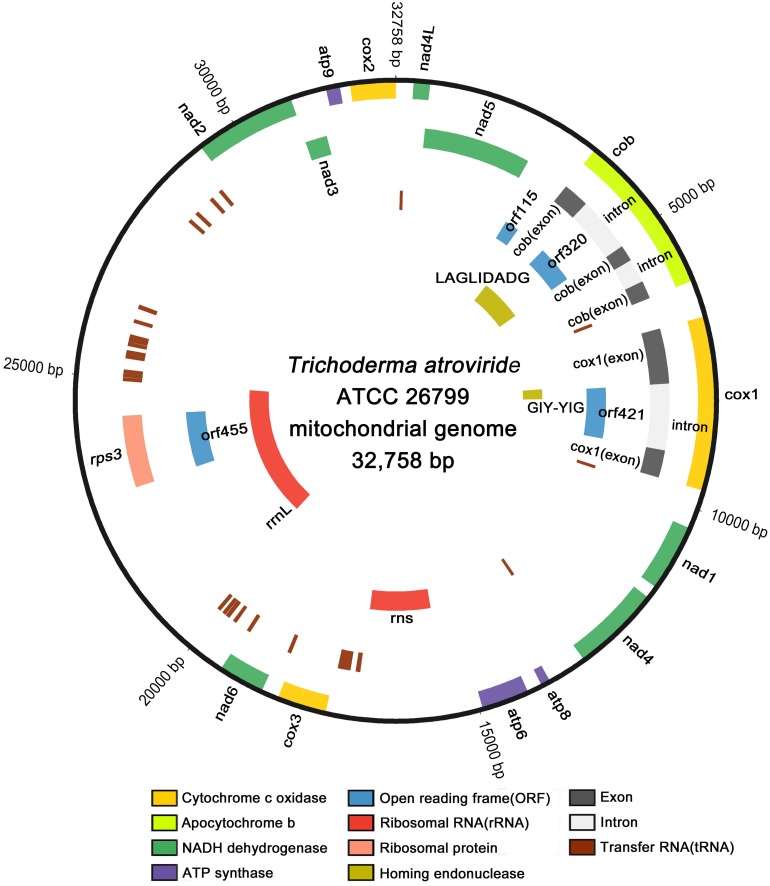
Circular map of the complete mitochondrial genome of *T. atroviride* ATCC 26799. All genes are located on the heavy strand (H-strand) and transcribed in the clockwise direction, except for one gene encoding the GIY-YIG-type homing endonuclease that located on the light strand (L-strand) and transcribed in the anticlockwise direction. Gene components were indicated with different color blocks, and the circular map was plotted using Circos.

Using the next-generation sequencing (NGS) of the genomic DNA from *Trichoderma atroviride* (formerly known as *T. harzianum*; Ban et al., 2010) ATCC 26799, the mitochondrial genome was assembled *de novo* with a high depth of coverage (314.81x; GenBank accession no. MN125601). The complete *de novo* mitochondrial genome was found to be a closed-circular DNA molecule with a length of 32,758 bp, consisting of 14 core protein-coding genes, four additional open reading frames (*orf* genes) encoding hypothetical proteins, 27 transfer RNA genes (tRNAs), two ribosomal RNA genes (rRNAs), one gene encoding the ribosomal protein S3, two genes harboring conserved domains for the homing endonuclease (HE) protein, and non-coding regions. All genes were located on the heavy strand (H-strand) and transcribed in a clockwise direction, except for one gene encoding the GIY-YIG-type HE protein located on the light strand (L-strand) and transcribed in an anticlockwise direction (Figure 1).

The overall nucleotide compositions of the complete mitochondrial genome was A (36.1%), T (35.7%), G (15.4%), and C (12.8%), meaning the proportion of AT content (71.8%) was ≈2.54 times higher than that of GC content (28.2%). The asymmetric bias of nucleotide composition was estimated based on the skewness, with the obtained values all positive (0.006 for AT-skew and 0.091 for GC-skew), meaning that adenine (A) and guanine (G) occurred more frequently than did thymine (T) and cytosine (C) in the H-strand. The non-coding regions ranged in size from 4 to 1,842 bp and accounted for ≈24.93% of the entire length of the mitochondrial genome (Table 1).

**TABLE 1:** Genomic organization of the *T. atroviride* ATCC 26799 mitochondrial genome.

**Gene**	**Strand^a^**	**Start position**	**Stop position**	**Length (bp)**	**Start codon**	**Stop codon**	**Note**
*nad4L*	H	292	561	270	ATG	TAA	
*nad5*	H	561	2,627	2,067	ATG	TAA	
*orf115*	H	2,881	3,228	348	ATG	TAA	Hypothetical protein
*cob*	H	3,498	6,182	2,685	ATG	TAA	*cob* CDS (3,498..4,004; 5,062…5,379; 5,835…6,182)
*cob*-intron 1	H	4,005	5,061	1,057	–	–	
*cob*-intron 2	H	5,380	5,834	455	–	–	
*orf320*	H	4,035	4,967	933	ATG	TAA	Hypothetical protein
*cox1*	H	6,801	9,672	2,872	ATG	TAA	*cox1* CDS (6,801…7,862; 9,142…9,672)
*cox1*-intron	H	7,863	9,141	1,279	–	–	
*orf421*	H	7,866	9,131	1,266	TTA	TAA	Hypothetical protein
*nad1*	H	10,326	11,435	1,110	ATG	TAA	
*nad4*	H	11,647	13,104	1,458	ATG	TAA	
*atp8*	H	13,763	13,909	147	ATG	TAA	
*atp6*	H	14,161	14,943	783	ATG	TAA	
*rns*	H	15,514	17,017	1,504	–	–	Small subunit rRNA
*cox3*	H	17,536	18,345	810	ATG	TAA	
*nad6*	H	18,638	19,390	753	ATG	TAA	Putative *trnV*^Val^
*rrnL*	H	20,245	24,937	4,693	–	–	Large subunit rRNA
*rps3*	H	22,890	24,278	1,389	ATT	TAA	Ribosomal protein S3
*orf455*	H	22,911	24,278	1,368	ATG	TAA	Hypothetical protein
*nad2*	H	29,342	31,009	1,668	ATG	TAA	
*nad3*	H	31,010	31,423	414	ATG	TAA	
*atp9*	H	31,610	31,834	225	ATG	TAA	
*cox2*	H	32,009	32,758	750	ATG	TAA	
Homing endonuclease	H	3,471	4,967	1,497	ATA	TAA	LAGLIDADG
	L	7,787	8,131	345	ATA	TAG	GIY-YIG

**Other features**					**Value**		

Mitochondrial genome size (bp)					32,758		
AT content (%)/GC content (%)					71.8 (A, 36.1%; T, 35.7%)/28.2 (G, 15.4%; C, 12.8%)		
AT-skew/GC-skew					0.006/0.091		
Intergenic region (%)^b^					24.93		
two rRNAs + 27 tRNAs (%)^b^					25.04		
GenBank accession no.					MN125601		

*^*a*^H, heavy strand (H-strand); L, light strand (L-strand). ^*b*^% of total nucleotide sequences (nt) of the mitochondrial genome.*

All 14 conserved core protein-coding genes were 16,012 bp in size and composed of three ATP synthases (*atp6*, *atp8*, and *atp9*), one apocytochrome *b* (*cob*), three cytochrome *c* oxidases (*cox1*, *cox2*, and *cox3*), and seven NADH dehydrogenases (*nad1*, *nad2*, *nad3*, *nad4*, *nad4L*, *nad5*, and *nad6*). Of these genes, *cox1* was the longest (2,872 bp) and the shortest was *atp8* (147 bp). Four open reading frames without predicted functions (*orf115*, *orf320*, *orf421*, and *orf455*) were also found. All core genes and ORFs were initiated with a standard start codon of ATG and terminated with the TAA stop codon, except for *orf421*, which had a TTA initiation codon. There was an overlap of one nucleotide base between the TAA stop codon of *nad4L* and the ATG start codon of *nad5* (-1 bp, corresponding to the codon A). Of the identified *orf* genes, *orf115* was determined to be a free-standing ORF, whereas *orf320* and *orf421* were located entirely within the introns of *cob* and *cox1*, respectively. The *orf455* gene was also found to be completely within the coding region of the *rps*3 gene for ribosomal protein S3 (Table 1).

The total length of the tRNA genes (*trn* genes) was 2,007 bp, accounting for ≈6.13% of the entire size of the mitochondrial genome. The average length of the tRNA genes ranged from 70 bp (*trnF*^Phe^) to 87 bp (*trnS*^Ser^), and all of the 27 tRNAs were predicted to be free-standing on the H-strand (Supplementary Table S1). Most of the tRNA genes were predicted to have a typical cloverleaf secondary structure, but some *trn* genes (*trnY*^Tyr^, *trnS*^Ser^, and *trnL*^Leu^) were found to display an extra variable arm (V-arm) (Supplementary Figure S1). Multiple copies of several of the *trn* genes were found with different anticodons. There were three copies of *trnR*^Arg^ for arginine (one copy with *trnR*^Arg^ [ACG] and two copies with *trnR*^Arg^[TCT]), two copies of *trnF*^Phe^ for phenylalanine (*trnF*^Phe^ [AAA] and *trnF*^Phe^[GAA]), two copies of *trnS*^Ser^ for serine (*trnS*^Ser^[GCT] and *trnS*^Ser^[TGA]) and two copies of *trnL*^Leu^ for leucine (*trnL*^Leu^[TAA] and *trnL*^Leu^[TAG]). Four copies of the *trnM*^Met^ gene coding for methionine were found with the same anticodon (*trnM*^Met^[CAT]) (Supplementary Table S1).

Two rRNA genes, the *rns* gene for small subunit ribosomal RNA and the *rrnL* gene for large subunit ribosomal RNA, were located on the H-strand with lengths of 1,504 bp and 4,693 bp, respectively. The *rns* gene was located between *atp6* and *cox3*, but the *rrnL* gene was detected in a position ranging over both *orf455* and *rps3*. Both *orf455* and *rps3* were positioned as internal ORFs, fully within the coding region of the *rrnL* gene (Figure 1 and Table 1). All of the 27 tRNA genes and the two rRNA genes accounted for 25.04% of the total length of the mitochondrial genome (Table 1).

In the complete mitochondrial genome of *T. atroviride* ATCC 26799, leucine (L) was the most frequent amino acid (with a relative frequency of 12.1%) and was encoded by six codons: TTA (46.2%), CTA (16.5%), CTT (14.9%), TTG (10.8%), CTG (6.3%), and CTC (5.4%). Then, isoleucine (I, 11.0%), tyrosine (Y, 7.1%), and serine (S, 6.9%) were the next most frequently occurring amino acids, while methionine (M) was the least common (1.7%) (Supplementary Table S2).

Through a self-comparison of the mitochondrial genome in BLAST searches, three interspersed repeat sequences were identified with lengths ranging from 39 to 56 bp. The largest repeats were found ca. 565 bp away from each other, harboring 95% similarity, and the intergenic region between these two repeat sequences contained five *trn* genes (*trnM*^Met^, *trnL*^Leu^, *trnA*^Ala^, *trnF*^Phe^, and *trnK*^Lys^). The shortest repeats were located in the region between the 3′ end region of the *atp6* gene and *rns* gene, harboring 100% similarity. These short repeat sequences were 357 bp apart from each other, but no gene was found between these two short repeats. Furthermore, four types of tandem repeats were detected with sizes ranging from 14 to 21 bp, and these repeats were presented in two- or three copies on the mitochondrial genome. The repeat sequences, which were mostly duplicated and 20 bp or 21 bp in size, were observed either in the coding region of the *nad4L* gene or in the intergenic region between *nad5* and *orf115*. However, all of the tandem repeats, of which there were three copies of sizes 14 bp or 15 bp, were found within the *orf455* gene surrounded by two genes, *rps3* and *rrnL* (Supplementary Table S3).

### Phylogenetic Analysis of the *T. atroviride* ATCC 26799 Mitochondrial Genome Within the Order *Hypocreales*

The taxonomic location of the *T. atroviride* ATCC 26799 mitochondrial genome was investigated based on the molecular phylogeny of Hypocreales. The publicly available mitochondrial genomes of *Hypocreales* species were retrieved from the GenBank database on NCBI, mitochondrial genomes harboring core genes in multiple copies were excluded to remove the potential effects of intra-genomic heterogeneity (Waterhouse et al., 2011; Hellmuth et al., 2015), 38 *Hypocreales* species were used in the phylogenetic analysis (Supplementary Table S4). One *Sordariales* strain (*Neurospora crassa* OR74A), belonging to the class Sordariomycetes, which also contains Hypocreales, was used as an outgroup.

**FIGURE 2:**
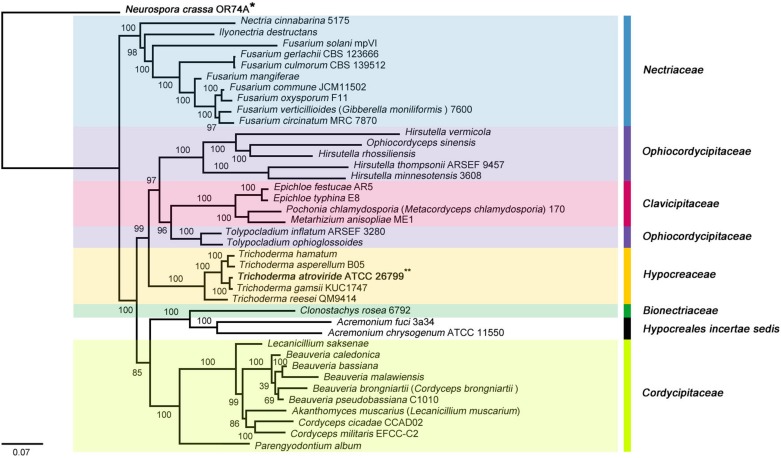
Phylogenetic tree of the *Hypocreales* species (Sordariomycetes) mitochondrial genomes based on ML (maximum likelihood) analysis. The tree was generated using concatenated sequences of 13 core genes (*atp6, atp8, cob, cox1*, *cox2*, *cox3*, *nad1*, *nad2*, *nad3*, *nad4*, *nad4L*, *nad5*, and *nad6*), and the mitochondrial genome of *Neurospora crassa* OR74A (Sordariales) was used as an outgroup. All *Sordariomycetes* species that used for the phylogenetic tree were described in Supplementary Table S4, and ML bootstrap (BS) values were marked on the nodes.

The molecular phylogenies were well supported with high confidences, and all 38 *Hypocreales* species were divided into seven major clades (Bionectriaceae, Clavicipitaceae, Cordycipitaceae, Hypocreaceae, Hypocreales incertae sedis, Nectriaceae, and Ophiocordycipitaceae). To the relatedness between the clades, the Hypocreaceae family, which includes the *Trichoderma* genus, was found to be a sister clade of Ophiocordycipitaceae/Clavicipitaceae and clustered as {[[(Hypocreaceae) + (Ophiocordycipitaceae + Clavicipitaceae)] + (Cordycipitaceae + Bionectriaceae + Hypocreales incertae sedis)] + Nectriaceae} on the ML- and BI tree. Among hypocrealean fungi, a sister relationship between *T. atroviride* ATCC 26799 and *T. gamsii* KUC1747 was observed identically in both tree topologies (*BS* = 100, BPP = 1.00) (Figure 2 and Supplementary Figure S2).

### Comparative Analysis of the Mitochondrial Genomes of *Trichoderma* Species

**FIGURE 3:**
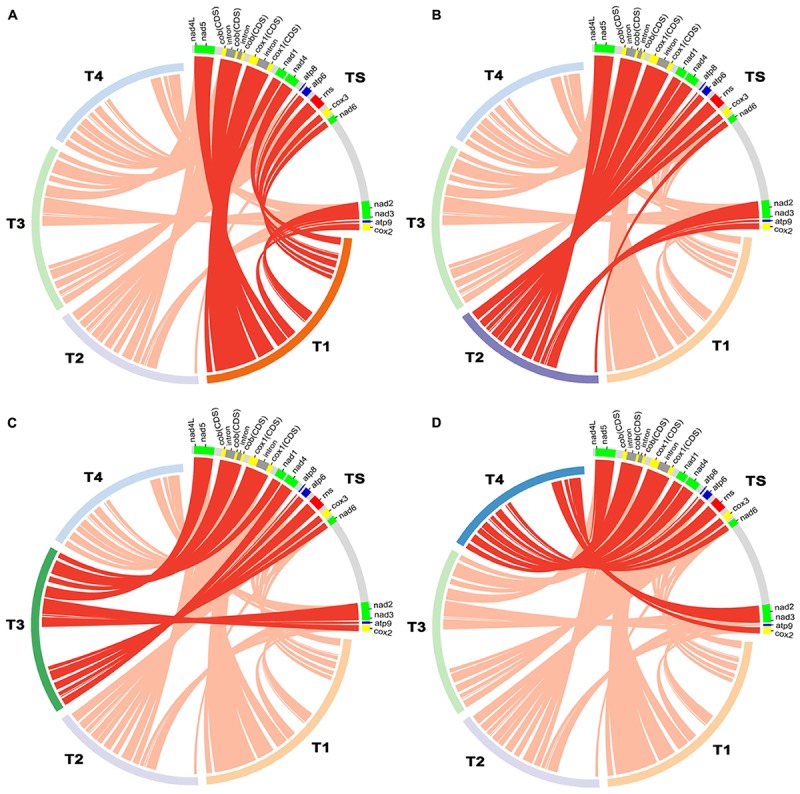
Comparative analysis of mitochondrial gene orders among *Trichoderma* species. Details of gene components (14 core genes and *rns* gene) used for the comparative analysis were described in Supplementary Table S5, and plots were generated using Circos. **(A)** compared between *T. atroviride* ATCC 26799 (TS) and *T. reesei* QM9414 (T1). **(B)** compared between *T. atroviride* ATCC 26799 (TS) and *T. asperellum* B05 (T2). **(C)** compared between *T. atroviride* ATCC 26799 (TS) and *T. hamatum* (T3). **(D)** compared between *T. atroviride* ATCC 26799 (TS) and *T. gamsii* KUC1747 (T4).

The complete mitochondrial genome of *T. atroviride* ATCC 26799 was compared with mitochondrial genomes from other *Trichoderma* species, such as *T. reesei* QM9414 (GenBank accession no. AF447590), *T. asperellum* B05 (GenBank accession no. NC_037075), *T. hamatum* (GenBank accession no. MF287973), and *T. gamsii* KUC1747 (GenBank accession no. KU687109) (Table 2). Synteny analysis revealed that the *Trichoderma* mitochondrial genomes could be divided into five representative regions and relatively positioned in various sizes across the mitochondrial genomes (Supplementary Figure S3). All of the core genes and *rns* gene were observed in a highly conservative gene orders for each synthenic blocks, and the mitochondrial genome of *T. atroviride* ATCC 26799 also exhibited this conservative gene arrangements (Figure 3).

**TABLE 2:** Features of *Trichoderma* mitochondrial genomes that used for comparative analyses.

**Strain**	***T. atroviride* ATCC 26799**	***T. reesei* QM9414^a^**	***T. asperellum* B05^a^**	***T. hamatum*^a^**	***T. gamsii* KUC1747^a^**
Mitochondrial genome size (bp)	32,758	42,130	29,999	32,763	29,303
AT content (%)	71.8	72.8	72.2	72.3	71.7
GC content (%)	28.2	27.2	27.8	27.7	28.3
AT-skew	(+) 0.006	(+) 0.041	(-) 0.066	(-) 0.002	(-) 0.062
GC-skew	(+) 0.091	(+) 0.086	(+) 0.043	(+) 0.031	(+) 0.036
Protein CDS	21	19	17	20	18
rRNA genes	2	2	1	2	2
tRNA genes^b^	27 (1)	24 (2)	25 (1)	26 (1)	26 (1)
Intergenic region (%)^c^	24.93	17.58	31.73	19.05	30.15
Genome identity (%)^d^	Used as query	94.16 (85.0%)	97.30 (82.0%)	96.26 (87.0%)	98.75 (88.0%)
GenBank accession no.	MN125601	AF447590	NC_037075	MF287973	KU687109
Note	This study	–	Non-existence of *rrnL* gene	–	Non-existence of *atp9* gene
In the Figures 3–5, indicated as	TS	T1	T2	T3	T4

*^*a*^Based on the GenBank database on NCBI (datasets as of, December 2019). ^*b*^A number of putative trn genes predicted within the protein-coding genes were indicated inside the parentheses. ^*c*^% of total nucleotide sequences (nt) of the mitochondrial genome. ^*d*^Using BLAST searches between T. atroviride ATCC 26799 and other Trichoderm species; values of query coverage (%) for the sequence identity were indicated inside the parentheses.*

There was a higher variability among *Trichoderma* species in terms of intergenic regions, nucleotide composition bias, number of protein CDS, and the total size of the mitochondrial genome. As shown in Table 2, the intergenic region ranged from 17.58% (*T. reesei* QM9414) to 31.73% (*T. asperellum* B05), with the mitochondrial genome of *T. atroviride* ATCC 26799 falling in the middle of this range. The proportion of nucleotide bases (AT- and GC) was similar, but the skewness value showed variability. The mitochondrial genomes of *T. asperellum* B05, *T. hamatum*, and *T. gamsii* KUC1747, showed negative values of AT-skew, reflecting more frequency of thymine (T) than adenine (A), while the value of AT-skew was positive for *T. atroviride* ATCC 26799. The gene sets for NADH dehydrogenases were of a similar size across the *Trichoderma* mitochondrial genomes, but the lengths of the genes coding for apocytochrome *b* (*cob*) and cytochrome *c* oxidases (*cox1*, *cox2*, and *cox3*) varied in size due to the presence of introns (Supplementary Table S5). The mitochondrial genome of *T. atroviride* ATCC 26799 carried more protein CDS and *trn* genes than those of other *Trichoderma* mitochondrial genomes. As a result of these variations, the overall size of the *Trichoderma* mitochondrial genomes ranged widely from 29,303 bp (*T. gamsii* KUC1747) to 42,130 bp (*T. reesei* QM9414), with the mitochondrial genome of *T. atroviride* ATCC 26799 falling in the middle of this range. Compared to the other *Trichoderma* mitochondrial genomes, *T. atroviride* ATCC 26799 showed mean sequence identity of 96.61% over 85.5% query coverage (Table 2).

**FIGURE 4:**
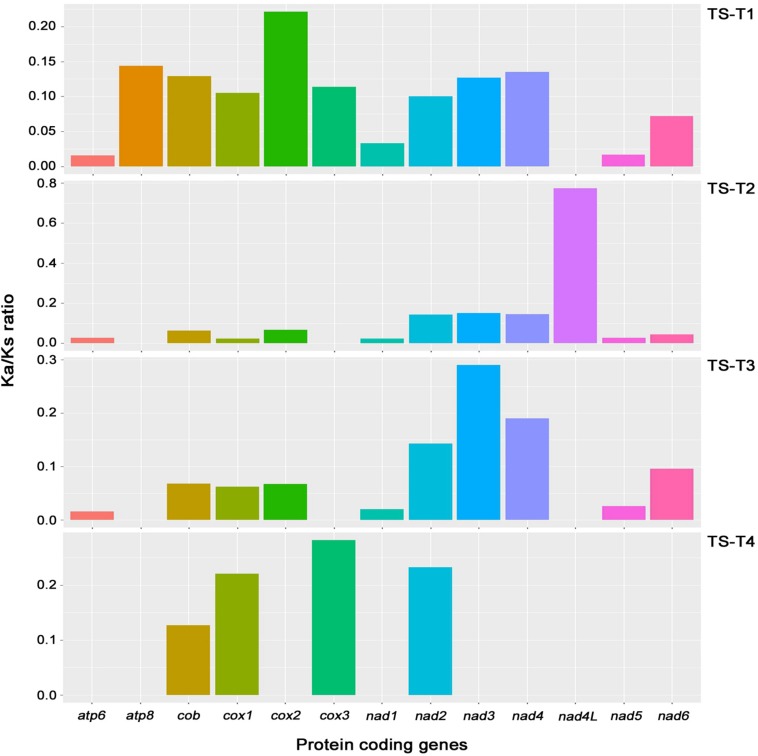
Analysis of Ka/Ks ratios for 13 core genes of the *T. atroviride* ATCC 26799 mitochondrial genome. Obtained values for Ka/Ks ratios were described in Supplementary Table S6. Ka, nonsynonymous substitution rate; Ks, synonymous substitution rate; TS-T1, pairwise alignment between *T. atroviride* ATCC 26799 (TS) and *T. reesei* QM9414 (T1); TS-T2, pairwise alignment between *T. atroviride* ATCC 26799 (TS) and *T. asperellum* B05 (T2); TS-T3, pairwise alignment between *T. atroviride* ATCC 26799 (TS) and *T. hamatum* (T3); TS-T4, pairwise alignment between *T. atroviride* ATCC 26799 (TS) and *T. gamsii* KUC1747 (T4).

On the comparisons with other *Trichoderma* species, the Ka/Ks ratio of 13 core genes (*atp6*, *atp8*, *cob*, *cox1*, *cox2*, *cox3*, *nad1*, *nad2*, *nad3*, *nad4*, *nad4L*, *nad5*, and *nad6*; the *atp9* gene was excluded because it was absent from the mitochondrial genome of *T. gamsii* KUC1747) were assessed in the mitochondrial genome of *T. atroviride* ATCC 26799. The highest ratio (0.774) was found for the *nad4L* gene, nevertheless, all of the Ka/Ks ratios were lower than one (<1), indicating that the core genes of the *T. atroviride* ATCC 26799 mitochondrial genome evolve under purifying selection (Figure 4 and Supplementary Table S6).

### Evolution of Mitochondrial Introns Among *Trichoderma* Species

**FIGURE 5:**
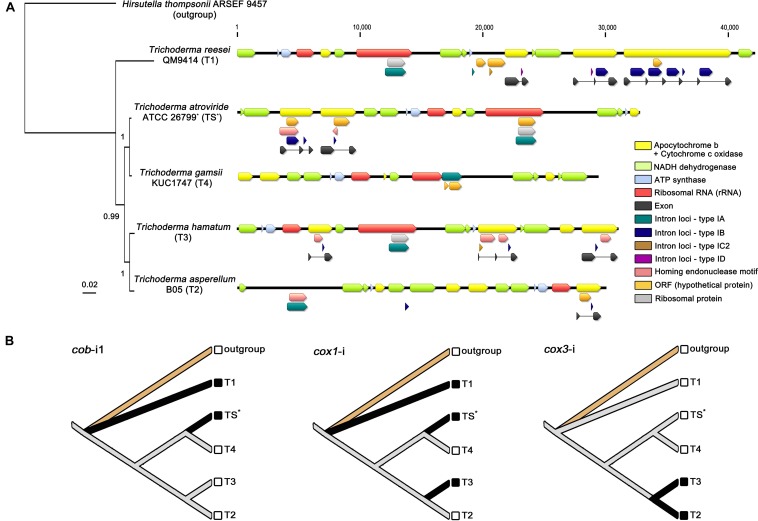
Comparative analysis of *Trichoderma* mitochondrial introns. **(A)** All detected intron loci (described in Table 3) were displayed on the mitochondrial genomes with a BI based topology constructed using concatenated exon nucleotide sequences of 13 core genes (*atp6, atp8, cob, cox1*, *cox2*, *cox3*, *nad1*, *nad2*, *nad3*, *nad4*, *nad4L*, *nad5*, and *nad6*). All gene components were indicated with different color blocks. **(B)** Inference of gain/loss events of *Trichoderma* mitochondrial introns. Reconstructed ancestral states for introns were presented with species names designated as, outgroup for *Hirsutella thompsonii* ARSEF 9457 (Ophiocordycipitaceae), TS for *T. atroviride* ATCC 26799 (with an asterisk), T1 for *T. reesei* QM9414, T2 for *T. asperellum* B05, T3 for *T. hamatum*, and T4 for *T. gamsii* KUC1747. Solid black lines indicate the presence of a given intron character in corresponding species.

In the fungal mitochondrial genomes, introns, capable of self-splicing, are widely known as one of factors leading structural variability of mitochondrial genomes (e.g., variation in gene content, the complexity of gene arrangements, and changes in genome size) (Hurst and Werren, 2001; Haugen et al., 2005; Lang et al., 2007). Introns can be classified into two categories, group I and group II depending on the intron sequences, the conserved secondary-/tertiary structures of intron RNA, and the splicing mechanisms (Lang et al., 2007). On the mitochondrial genome of *T. atroviride* ATCC 26799, four introns, all belonging to the group I, were predicted. Of these introns, three (all classified in the subtype of IB) were observed in the exon-intron structures of protein-coding genes for apocytochrome *b* (*cob*) and cytochrome *c* oxidases (*cox1*), and the remaining one (classified in the subtype of IA) was found in the *rrnL* gene. These introns were also predicted to harbor an internal ORF within the region of intron themselves. The *rps3* gene of the *T. atroviride* ATCC 26799 mitochondrial genome was predicted to be an internal ORF of *rrnL-i* (intron that was fully embedded within the *rrnL* gene), which is consistent with previous reports describing the intron-encoded *rps3* gene in fungal mitochondrial genomes (Lang et al., 2007; Sethuraman et al., 2009; Wai et al., 2019). The 1,497 bp ORF harboring the LAGLIDADG motif for the HE protein (79.31% identity with that of *Fusarium graminearum* (GenBank accession no. YP_001249321) under a 63% query coverage; *E*-value = 7e-161) was located in the *cob* gene, overlapping the coding region and *cob-i*1 (first intron in the exon-intron structure of the *cob* gene). A 345 bp ORF sharing a homology with the GIY-YIG motif of the HE protein (65.42% identity with that of *F. acuminatum* (GenBank accession no. CDL73454) under a 93% query coverage; *E*-value = 5e-34) was also observed on the L-strand, as an internal ORF of *cox1-i* (intron positioned in the *cox1* gene). These two HE proteins were initiated with the ATA codon. Like the other protein-coding genes, the LAGLIDADG-type HE protein was terminated with a TAA codon, but the GIY-YIG-type HE protein had a TAG stop codon. Lastly, of the four introns, *cob-i*2 (second intron in the exon-intron structure of the *cob* gene) did not have an internal ORF (Tables 1, 3 and Figure 5A).

**TABLE 3:** Detected group I intron loci on the mitochondrial genomes of *Trichoderma* species.

**Species (GenBank assession no.)**	**Total number of introns (Numbers of subtypes)**	**Replacement**			
		**Subtype**	**Start position – Stop position [Length (nt)]^b^**	**Open reading frame^*c*^**	**Note**
*T. atroviride* ATCC 26799 (MN125601, this study)	4 [IA (1), IB (3)]	IA IB IB IB	22,709–24,328 (1,620 bp) 4,042–4,989 (948 bp) 5,404–5,638 (235 bp) 7,889–8,068 (180 bp)	*rps3*, *orf455 orf320*, LAGLIDADG – *orf421*, GIY-YIG	*rrnL-i cob-i*1 *cob-i*2 *cox1-i*
*T. reesei* QM9414 (AF447590)^a^	11 [IA (2), IB (6), C2 (1), ID (2)]	IA IA IB IB IB IB IB IB IC2 ID ID	12,058–13,712 (1,655 bp) 19,142–19,317 (176 bp) 29,238–30,185 (948 bp) 32,036–33,177 (1,142 bp) 33,521–34,590 (1,070 bp) 34,992–36,015 (1,024 bp) 36,298–36,477 (180 bp) 37,672–38,712 (1,041 bp) 20,553–20,784 (232 bp) 23,141–23,290 (150 bp) 28,844–28,966 (123 bp)	*rps5 orf* – – *orf* – – – *orf* – –	*rrnL-i* – *cob-i*2 *cox1-i*1 *cox1-i*2 *cox1-i*3 *cox1-i*4 *cox1-i*5 – *cox2-i cob-i*1
*T. asperellum* B05 (NC_037075)^a^	3 [IA (1), IB (2)]	IA IB IB	4,056–5,686 (1,631 bp) 13,682–13,968 (287 bp) 28,833–28,979 (147 bp)	*orf459* – *orf305*	putative *rrnL-i* – *cox3-i*
*T. hamatum* (MF287973)^a^	5 [IA (1), IB (3), IC2 (1)]	IA IB IB IB IC2	12,404–14,023 (1,620 bp) 6,995–7,141 (147 bp) 22,120–22,306 (187 bp) 29,220–29,399 (180 bp) 19,786–20,017 (232 bp)	*rps3* LAGLIDADG GIY-YIG GIY-YIG LAGLIDADG	*rrnL-i cox3-i cox2-i*2 *cox1-i cox2-i*1
*T. gamsii* KUC1747 (KU687109)^a^	1 [IA (1)]	IA	16,622–18,230 (1,609 bp)	Two *orf* genes	Both for *rrnL-i*

*^*a*^Based on the GenBank database on NCBI (datasets as of, December 2019). ^*b*^Descriptions of the position and sizes of intron loci detected on the RNAweasel webserver (http://megasun.bch.umontreal.ca/cgi-bin/RNAweasel/RNAweaselInterface.pl). ^*c*^Intronic ORF (i.e., intron-encoded ORF); rps3/rps5, genes coding for ribosomal proteins; orf, gene coding for hypotethical protein; LAGLIDADG or GIY-YIG, conserved structural motifs of homing endonuclease (HE); Non-existence of genes were indicated with a dash (–).*

A total number of 24 intron loci was detected in all five *Trichoderma* mitochondrial genomes, 11 of which were found in the *T. reesei* QM9414, whereas only one of the intron locus was presented in the *T. gamsii* KUC1747 (Table 3). Most of intron sites were detected in the exon-intron structures of protein-coding genes for apocytochrome *b* (*cob*) and cytochrome *c* oxidases (*cox1*, *cox2*, and *cox3*), but not in the core genes for ATP synthases (*atp6*, *atp8*, and *atp9*) and NADH dehydrogenases (*nad1*, *nad2*, *nad3*, *nad4*, *nad4L*, *nad5*, and *nad6*). A group IA-type intron (i.e., an intron classified as the subtype IA) was observed within the region of the *rrnL* gene in every *Trichoderma* species except *T. asperellum* B05 (in this study, the possibility of the *rrnL* gene being present in the *T. asperellum* B05 mitochondrial genome was excluded because this gene has not been reported in the mitochondrial genome of this species). This broad existence of *rrnL-i* may indicate that it was present in the ancestor of the genus *Trichoderma* but, at the same time, the various intronic ORFs detected within the *rrnL-i* (e.g., *rps3*, *rps5*, and *orf* genes without predicted functions) suggest there has been a divergence in *rrnL-i* among the *Trichoderma* species. Interestingly, unlike the other *Trichoderma* species, the mitochondrial genome of *T. atroviride* ATCC 26799 was found to have multiple intronic ORFs in the intron region, with the group IA-type *rrnL-i* harboring two intronic ORFs (*rps3* and *orf455*), the group IB-type *cob-i*1 with both *orf320* and ORF for the LAGLIDADG-type HE protein, and the group IB-type *cox1-i* harboring *orf421* and ORF for the GIY-YIG-type HE protein. In the mitochondrial genome of *T. atroviride* ATCC 26799, both *cox2* and *cox 3* were identified as intronless genes (Table 3 and Figure 5A).

To estimate the evolution of mitochondrial introns, a phylogenetic tree using the concatenated exon nucleotide sequences of 13 core genes (*atp6*, *atp8*, *cob*, *cox1*, *cox2*, *cox3*, *nad1*, *nad2*, *nad3*, *nad4*, *nad4L*, *nad5*, and *nad6*; the *atp9* gene was excluded because it was absent from the mitochondrial genome of *T. gamsii* KUC1747) was constructed with the outgroup *Hirsutella* thompsonii ARSEF 9457 (Hypocreales, Ophiocordycipitaceae). In the obtained exon phylogeny, a clustering of all five *Trichoderma* species was supported with high confidences (BPP ≥ 0.99, with an average standard deviation for split frequencies of <0.01) (Figure 5A), this BI based topology followed the same pattern as those shown in the phylogeny of Hypocreales using concatenated full nucleotide sequences (including all of the exon-intron structures) of the 13 core genes (Supplementary Figure S2). This result suggests that the observed introns of mitochondrial core genes may share a common evolutionary history with the core protein-coding genes in these five *Trichoderma* species.

Several gain/loss events for the introns were also traced using a reconstruction of the ancestral states of the introns. For instance, *cob-i*1 (i.e., meaning as a homologous intron of the *cob-i*1 in *T. atroviride* ATCC 26799) seems to have been gained twice and lost at least once, whereas *cox1-i* (i.e., meaning as an orthologous intron of the *cox1-i* in *T. atroviride* ATCC 26799) is thought to have been gained at least three times and lost once in *Trichoderma s*pecies. Interestingly, *cox3-i* (i.e., meaning as an orthologous intron of the *cox3-i* in *T. hamatum*) seems to have experienced only with the gain event once (Figure 5B).

The four *Trichoderma* species that split from a common ancestor node of *T. reesei* QM9414 were shown to have fewer introns than *T. reesei* QM9414. These introns were also observed to be short, and some did not have an intronic ORF in the region of the intron (Figure 5A). These changes in the introns may be an indication of the ongoing degeneration of these introns in *Trichoderma* species. Finally, as shown in the sequence similarity for LAGLIDADG-/GIY-YIG-type HE proteins between *T. atroviride* ATCC 26799 and *Fusarium* species (Hypocreales, Nectriaceae), the *rps3* gene of the *T. atroviride* ATCC 26799 mitochondrial genome had a high sequence identity of 96.61% with that of *T. hamatum* (under a 94% query coverage; *E*-value = 0.0). These results also support the horizontal transfer of introns between the inter- and intraspecific diversity of fungi.

## Discussion

The mitochondrial ribosomal protein S3, which contributes to the assembly of the mitochondrial small ribosomal subunit and commonly assumed to be one of ancient genes related to the evolution of mitochondrial genomes in eukaryotes (Korovesi et al., 2018), has been known as the only ribosomal protein encoded by fungal mitochondrial genomes (Bullerwell et al., 2000). The *rps3* gene that codes for ribosomal protein S3 is usually reported to be a free-standing gene or an internal ORF of group I intron positioned within the *rrnL* gene coding for large subunit ribosomal RNA (Sethuraman et al., 2009; Chan and Lowe, 2019; Wai et al., 2019). In the mitochondrial genome of *T. atroviride* ATCC 26799, the *rps*3 gene was predicted to be 1,389 bp (21 bp larger than *orf455*) with an ATT initiation codon and was observed in the *rrnL-i*. When compared with average lengths of ribosomal protein S3 in *Hypocreales* species, which has previously been reported to be 376–514 amino acids long (Korovesi et al., 2018), this protein was found to fall in the middle of this range in the mitochondrial genome of *T. atroviride* ATCC 26799, with a length of 463 amino acids (Figure 1 and Table 1).

In the mitochondrial genome of *T. atroviride* ATCC 26799, some *trn* genes (*trnY*^Tyr^, *trnS*^Ser^, and *trnL*^Leu^) were found to display an extra variable arm (V-arm) (Supplementary Figure S1). The V-arm in the tRNA secondary structure is known to be specific to tRNA^Leu^, tRNA^Ser^, and bacterial-/eukaryotic organellar tRNA^Tyr^ (Fujishima and Kanai, 2014; Hamashima et al., 2016). All of the *trn* genes predicted to harbor a V-arm structure on the *T. atroviride* ATCC 26799 mitochondrial genome were consistent with these previous reports. Interestingly, a putative *trnV*^*Val*^ gene, which overlapped completely with the 3′ end region of the *nad6* gene, was predicted in the mitochondrial genome. As with *T. atroviride* ATCC 26799, a *trn* gene fully integrated into the protein-coding genes has also been observed in other *Trichoderma* species. The *trnV*^*Val*^ gene, which entirely overlapped with the 3′ end region of the *nad6* gene, was identically found in the mitochondrial genomes of *T. reesei* QM9414, *T. asperellum* B05, *T. hamatum*, and *T. gamsii* KUC1747. In addition, the mitochondrial genome of *T. reesei* QM9414 was found to harbor one, the *tRNA*^Met^ gene completely integrated within the 5′ end coding region of the *nad2* gene (Supplementary Table S7). In terms of tRNA genes that fully and/or partially overlap with protein-coding genes in the mitochondrial genomes, several studies of metazoan-/nematode mitochondrial genomes have suggested that it allows 1) avoiding the loss of tRNA genes when small genome size is selected, and/or 2) for keeping gene functions by the co-evolution of the two overlapping genes in compacted mitochondrial genomes (Fujishima and Kanai, 2014; Hamashima et al., 2016). Although expression mechanisms of tRNAs, that overlapped in functional mRNAs of protein-coding genes, have not yet been fully understood (Doublet et al., 2015), nevertheless, all these putative *trn* genes predicted in the protein-coding genes (*trnV*^*Val*^ and *tRNA*^Met^) seem to strongly support a complex correlation between tRNAs distributions and dynamic evolutions of mitochondrial genomes in *Trichoderma*.

A number of previous studies on the phylogenetic relationships of *Sordariomycetes* mitochondrial genomes have already shown that the phylogenetic replacements of Hypocreaceae varies in terms of clustering with related species. For example, in the study of the mitochondrial genome of the nematophagous fungus *Pochonia chlamydosporia* (Hypocreales, Sordariomycetes), Hypocreaceae was grouped as [(Hypocreaceae + Nectriaceae: *BS* = 92) + Clavicipitaceae: *BS* = 93] in ML analysis (Lin et al., 2015) while, in a study of the mitochondrial genome of the medicinal fungus *Ophiocordyceps sinensis* (Hypocreales, Sordariomycetes), a ML based tree was constructed with [(Hypocreaceae + Clavicipitaceae: *BS* = 77) + Cordycipitaceae: *BS* = 68] (Li et al., 2015). Similarly, in a study of the mitochondrial genome for *Hypomyces aurantius* (Hypocreales, Sordariomycetes), Hypocreaceae was clustered as [(Hypocreaceae + Clavicipitaceae: *BS* = 40) + Cordycipitaceae: *BS* = 52] in ML analysis (Deng et al., 2016). Meanwhile, as clustered in the Cordycipitaceae clade on the ML- and BI tree (Figure 2 and Supplementary Figure S2), a study of the mitochondrial genome for the endophytic fungus *Pestalotiopsis fici* (Xylariales, Sordariomycetes) showed a ML based phylogenetic tree in which some of the internal nodes could not be fully supported (*BS* = 59–72), though they were well supported in a BI tree (BPP = 1.00) (Zhang et al., 2017). Although differences in the analysis conditions (e.g., variations in the concatenated sequences and differences between the tree algorithms) need to be considered when comparing these previous studies, it is clear that informative datasets of the mitochondrial genomes from closely related family species are still necessary to improve the resolution of the *Hypocreales* phylogeny. From this perspective, the phylogenetic results obtained for the *T. atroviride* ATCC 26799 mitochondrial genome in the present study can be used to clarify the evolutionary relationships of the *Hypocreaceae* mitochondrial genomes with related *Hypocreales* species in Sordariomycetes.

To trace the gain/loss events for the introns, the ancestral states of these introns were reconstructed using the homologous introns of the *Trichoderma* mitochondrial genomes (Figure 5B). When the introns were compared in BLAST searches (*E*-values < 10^–10^), *cob-i*1 in *T. atroviride* ATCC 26799 was similar to *cob-i*2 in *T. reesei* QM9414 (88.73% identity under a 99% query coverage; *E*-value = 0.0), furthermore, *cox3-i* (intron positioned in the *cox3* gene) in *T. asperellum* B05 was similar to that of *T. hamatum* (97.12% identity under a 99% query coverage; *E*-value = 0.0). Although the *cox1* gene in *T. reesei* QM9414 was disrupted by five introns (all belonging to the same subtype IB), interestingly, only one *cox1-i*4 (fourth intron positioned in the *cox1* gene) exhibited a sequence similarity with *cox1-i* in *T. atroviride* ATCC 26799 (93.67% identity under a 96% query coverage; *E*-value = 0.0). None of the *cox2-i* introns (intron positioned in the *cox2* gene) had any similarities between them. The unique features of the introns, such as multiple intron sites in the same gene locus (e.g., five *cox1-i* in *T. reesei* QM9414) and/or the co-existences of different types of introns within a single coding gene (e.g., *cob* gene in *T. reesei* QM9414 harboring both subtype-IB and ID; *cox2* gene in *T. hamatum* harboring both subtype-IB and IC2), should be studied further to supprot the evolutionary history of *Trichoderma* mitochondrial introns fully reflecting intron dynamics. Nevertheless, the greatest significance of the results for the introns obtained in this study is that this represents the first comparative analysis of *Trichoderma* mitochondrial introns from an evolutionary perspective.

Recently, a time-scaled phylogenetic analysis was conducted to prove the genomic evolution of *Trichoderma* species using 638 core orthologous proteins from *Hypocreales* nuclear genomes. It showed that *Trichoderma* had diversified into three phylogenetically distant subclades ≈67 Mya. On the generated Bayesian chronogram, *T. harzianum* and *T. reesei* were clustered separately as different subclades, whereas *T. asperellum*, *T. hamatum*, *T. gamsii* and *T. atroviride* were clustered together in the same clade (Kubicek et al., 2019). This chronogram is not congruent completely with the *Trichoderma* phylogeny generated using the mitochondrial genomes. Unlike the position of *T. asperellum* as a basal group within the subclade of chronogram, a sister relationship between *T. asperellum* and *T. hamatum* was predicted in the present study (Figures 2, 5 and Supplementary Figure S2). This conflict seems to be associated with mitochondria evolutions, which has occurred independently of the nuclear genomes.

Since the genome sequence of *T. reesei* QM6a (GenBank accession no. AAIL00000000) was first reported (Martinez et al., 2008), researches involving the whole-genome sequencing of *Trichoderma* species have been increased to understand the biological mechanisms and evolutionary relationships of *Trichoderma* species. However, these studies have focused on the nuclear genomes (Kubicek et al., 2011, 2019; Mukherjee et al., 2013; Druzhinina et al., 2018). Among *T. harzianum* species, a 2.6 kb circular plasmid referred to as pThr1 containing only one CDS (designated as a putative reverse transcriptase) has been reported for the *T. harzianum* strain T95 (Antal et al., 2002). However, when compared with all five *Trichoderma* complete mitochondrial genomes (using BLAST searches: *E*-values < 10^–10^), there was no similarity between the plasmid sequences of pThr1 and these *Trichoderma* complete mitochondrial genomes, thus, the plasmid pThr1 may have been generated from other putative extraplasmids.

To date, very little information has been available on the whole sequences of *Trichoderma* mitochondrial genomes, in addition, there has been no attempt to characterize and comparatively analyze these *Trichoderma* mitochondrial genomes. In the present study, the complete mitochondrial genome of *T. atroviride* ATCC 26799 was assembled *de novo*, and its genomic features were characterized for the first time. Besides, it is the first report of comparative analysis of genomic features of *Trichoderma* mitochondrial genomes and also of tracing the footprints of specific genetic features that are likely to be associated with the evolution of the mitochondrial genomes of *Trichoderma* species. The results obtained in this study are useful for fully understanding *T. atroviride* as both a mycoparasite and a degrader of chemical compounds. Furthermore, they also contribute to elucidating the evolutionary processes underlying the *Trichoderma* mitochondrial genomes, thus offering further insight into the development of more effective fungal biocontrol agents.

## Data Availability Statement

The complete *de novo* mitochondrial genome sequence of *T. atroviride* ATCC 26799 [=IFO/NBRC 30543; formerly known as *T. harzianum* (Ban et al., 2010)] has been deposited in GenBank under the accession number MN125601.

## Author Contributions

YK performed all studies related with this research (designs of the project, performances of experiments, data analysis, and preparation of the manuscript).

## Conflict of Interest

The authors declare that the research was conducted in the absence of any commercial or financial relationships that could be construed as a potential conflict of interest.

## Footnotes

1 http://mitos.bioinf.uni-leipzig.de/index.py

2 https://megasun.bch.umontreal.ca/cgi-bin/dev_mfa/mfannotInterface.pl

3 http://megasun.bch.umontreal.ca/cgi-bin/RNAweasel/RNAweaselInterface.pl

4 www.ncbi.nlm.nih.gov/genbank/
