# Supplementary material for: Complete Mitochondrial Genome of the Fungal Biocontrol Agent Trichoderma atroviride: Genomic Features, Comparative Analysis and Insight Into the Mitochondrial Evolution in Trichoderma
Source: Front Microbiol. 2020 Apr 28;11:785. doi: 10.3389/fmicb.2020.00785 (PMC7228111; doi:10.3389/fmicb.2020.00785)
Supplement: Supplementary file 1 [file Data_Sheet_1.docx]

**SUPPLEMENTARY FIGURE LEGENDS**

**SUPPLEMENTARY FIGURE S1 |** Predicted secondary structure of transfer RNA genes (*trn* genes) that identified in the *T. atroviride* ATCC 26799 mitochondrial genome. All tRNA genes were indicated with numbers corresponding nucleotide positions in the mitochondrial genome. A putative *trnV*^Val^ gene that predicted fully within the coding region of the *nad6* gene was indicated by an asterisk.

**SUPPLEMENTARY FIGURE S2 |** Phylogenetic tree of the *Hypocreales* species (Sordariomycetes) mitochondrial genomes based on Bayesian inference (BI) analysis. The tree was generated using concatenated sequences of 13 core genes (*atp6, atp8,* *cob,* *cox1*, *cox2*, *cox3*, *nad1*, *nad2*, *nad3*, *nad4*, *nad4L*, *nad5*, and *nad6*), and the mitochondrial genome of *Neurospora crassa* OR74A (Sordariales) was used as an outgroup. All *Sordariomycetes* species that used for the phylogenetic tree were described in **Supplementary Table S4**. Bayesian Posterior Probabilities (BPP) values were marked on the nodes.

**SUPPLEMENTARY FIGURE S3 |** Synteny analysis of the *Trichoderma* mitochondrial genomes. The sequence alignments for syntenic blocks were run using Mauve (v.2.4.0) (Darling et al., 2010). All five representative blocks were indicated with different colors as follows: Dark green (block1), region containng *nad4L* and *nad5*; Blue (block 2), region containing *cob* and *cox1*; Purple (block 3), region contating *nad1*; Red (block 4), region containing *nad4*, *atp8*, *atp6*, *rns*, and *cox3*; Green (block5), region contating *nad6*, *rrnL*, *nad2*, *nad3*, *atp9*, and *cox2*. TS, mitochondrial genome of the *T. atroviride* ATCC 26799; T1, mitochondrial genome of the *T. reesei* QM9414; T2, mitochondrial genome of the *T. asperellum* B05; T3, mitochondrial genome of the *T. hamatum*; T4, mitochondrial genome of the *T. gamsii* KUC1747.
