# Supplementary material for: Complete Mitochondrial Genome of the Fungal Biocontrol Agent Trichoderma atroviride: Genomic Features, Comparative Analysis and Insight Into the Mitochondrial Evolution in Trichoderma
Source: Front Microbiol. 2020 Apr 28;11:785. doi: 10.3389/fmicb.2020.00785 (PMC7228111; doi:10.3389/fmicb.2020.00785)
Supplement: Supplementary file 2 [file Data_Sheet_2.docx]

**SUPPLEMENTARY TABLE S1 |** Transfer RNA genes that identified in the mitochondrial genome of *T. atroviride* ATCC 26799.

| Gene (product) | Strand^a^ | Start position | Stop position | Lenth (nt) | Anticodon |
| --- | --- | --- | --- | --- | --- |
| *trnR* (trnR-Arg) | H | 101 | 171 | 71 | ACG |
| *trnC* (trnC-Cys) | H | 6,236 | 6,307 | 72 | GCA |
| *trnR* (trnR-Arg) | H | 9,830 | 9,900 | 71 | TCT |
| *trnF* (trnF-Phe) | H | 13,261 | 13,330 | 70 | AAA |
| *trnY* (trnY-Tyr) | H | 17,061 | 17,144 | 84 | GTA |
| *trnD* (trnD-Asp) | H | 17,253 | 17,326 | 74 | GTC |
| *trnS* (trnS-Ser) | H | 17,332 | 17,415 | 84 | GCT |
| *trnN* (trnN-Asn) | H | 17,420 | 17,491 | 72 | GTT |
| *trnG* (trnG-Gly) | H | 18,432 | 18,502 | 71 | TCC |
| *trnI* (trnI-Ile) | H | 19,629 | 19,700 | 72 | GAT |
| *trnS* (trnS-Ser) | H | 19,790 | 19,876 | 87 | TGA |
| *trnW* (trnW-Trp) | H | 19,885 | 19,956 | 72 | TCA |
| *trnP* (trnP-Pro) | H | 20,010 | 20,081 | 72 | TGG |
| *trnT* (trnT-Thr) | H | 24,945 | 25,015 | 71 | TGT |
| *trnE* (trnE-Glu) | H | 25,021 | 25,093 | 73 | TTC |
| *trnM* (trnM-Met) | H | 25,094 | 25,164 | 71 | CAT |
| *trnM* (trnM-Met) | H | 25,381 | 25,453 | 73 | CAT |
| *trnL* (trnL-Leu) | H | 25,458 | 25,540 | 83 | TAA |
| *trnA* (trnA-Ala) | H | 25,623 | 25,694 | 72 | TGC |
| *trnF* (trnF-Phe) | H | 25,700 | 25,772 | 73 | GAA |
| *trnK* (trnK-Lys) | H | 25,773 | 25,845 | 73 | TTT |
| *trnM* (trnM-Met) | H | 26,076 | 26,146 | 71 | CAT |
| *trnL* (trnL-Leu) | H | 26,354 | 26,436 | 83 | TAG |
| *trnR* (trnR-Arg) | H | 28,279 | 28,351 | 73 | TCT |
| *trnQ* (trnQ-Gln) | H | 28,475 | 28,547 | 73 | TTG |
| *trnH* (trnH-His) | H | 28,861 | 28,934 | 74 | GTG |
| *trnM* (trnM-Met) | H | 29,094 | 29,165 | 72 | CAT |
| *trnV* (trnV-Val)^b^ | H | 19,308 | 19,379 | 72 | TAC |

^a^H, heavy strand (H-strand).

^b^A putative *trn* gene predicted within the coding region of the *nad6* gene.

**SUPPLEMENTARY TABLE S2 |** Codon usage in the mitochondrial genome of *T. atroviride* ATCC 26799.

| Amino acid  (AA) | | Codon  (% of AA) | Frequency value (%)^a^ | Amino acid (AA) | | Codon  (% of AA) | Frequency value (%)^a^ |
| --- | --- | --- | --- | --- | --- | --- | --- |
| A | Ala | GCA (30.2) | 2.3 | P | Pro | CCA (31.0) | 2.1 |
|  |  | GCC (15.5) |  |  |  | CCC (13.3) |  |
|  |  | GCG (6.3) |  |  |  | CCG (10.2) |  |
|  |  | GCT (48.0) |  |  |  | CCT (45.6) |  |
| C | Cys | TGC (35.3) | 2.1 | Q | Gln | CAA (59.6) | 2.5 |
|  |  | TGT (64.7) |  |  |  | CAG (40.4) |  |
| D | Asp | GAC (24.0) | 2.3 | R | Arg | AGA (42.2) | 3.9 |
|  |  | GAT (76.0) |  |  |  | AGG (28.2) |  |
| E | Glu | GAA (70.6) | 3.2 |  |  | CGA (9.5) |  |
|  |  | GAG (29.4) |  |  |  | CGC (4.0) |  |
| F | Phe | TTC (30.5) | 5.8 |  |  | CGG (7.3) |  |
|  |  | TTT (69.5) |  |  |  | CGT (8.8) |  |
| G | Gly | GGA (35.7) | 3.0 | S | Ser | AGC (13.7) | 6.9 |
|  |  | GGC (13.8) |  |  |  | AGT (28.0) |  |
|  |  | GGG (14.7) |  |  |  | TCA (17.6) |  |
|  |  | GGT (35.7) |  |  |  | TCC (7.9) |  |
| H | His | CAC (29.9) | 2.1 |  |  | TCG (6.5) |  |
|  |  | CAT (70.1) |  |  |  | TCT (26.3) |  |
| I | Ile | ATA (49.5) | 11.0 | T | Thr | ACA (35.6) | 4.2 |
|  |  | ATC (12.3) |  |  |  | ACC (16.2) |  |
|  |  | ATT (38.3) |  |  |  | ACG (14.7) |  |
| K | Lys | AAA (68.0) | 6.4 |  |  | ACT (33.6) |  |
|  |  | AAG (32.0) |  | V | Val | GTA (39.0) | 5.8 |
| L | Leu | CTA (16.5) | 12.1 |  |  | GTC (9.1) |  |
|  |  | CTC (5.4) |  |  |  | GTG (15.6) |  |
|  |  | CTG (6.3) |  |  |  | GTT (36.3) |  |
|  |  | CTT (14.9) |  | W | Trp | TGA (65.0) | 2.2 |
|  |  | TTA (46.2) |  |  |  | TGG (35.0) |  |
|  |  | TTG (10.8) |  | Y | Tyr | TAC (25.0) | 7.1 |
| M | Met | ATG (100) | 1.7 |  |  | TAT (75.0) |  |
| N | Asn | AAC (24.3) | 5.8 | STOP | TER | TAA (62.4) | 7.6 |
|  |  | AAT (75.7) |  |  |  | TAG (37.6) |  |

^a^Relative percentage (%) of total frequencies of expected codons

**SUPPLEMENTARY TABLE S3 |** Identification of repetitive elements in the mitochondrial genome of *T. atroviride* ATCC 26799.

| Interspersed repeat sequences detected by BLAST searches of the *T. atroviride* ATCC 26799 mitochondrial genome against itself (self-comparisons) | | | | | | | | | |
| --- | --- | --- | --- | --- | --- | --- | --- | --- | --- |
| Matching  no. | Identities (%) | E-value | | Mismatches  / Gaps | | Repeat element position as a Query  (Start position - Strop position,  Length (nt)) | | Repeat element position as a Subject  (Start position - Strop position,  Length (nt)) | |
| 1 | 95 | 4e-18 | | 3 / 0 | | 25,905 - 25,960  (56) | | 25,285 - 25,340  (56) | |
| 2 | 96 | 5e-17 | | 2 / 0 | | 28,785 - 28,835  (51) | | 28,488 - 28,538  (51) | |
| 3 | 100 | 3e-13 | | 0 / 0 | | 15,263 - 15,301  (39) | | 14,868 - 14,906  (39) | |
| Tandem repeat sequences detected in the mitochondrial genome of *T. atroviride* ATCC 26799 | | | | | | | | | |
| Matching  no. | Indices^a^ | | Consensus size^b^ | | Copy number^c^ | | Matches / Mismatches / Indels (%)^d^ | | Consensus repeat pattern |
| 1 | 362 - 400 | | 20 | | 1.9 | | 85.0 / 0.0 / 15.0 | | TAATGCTATTATCTATAAAA |
| 2 | 2,617 - 2,658 | | 21 | | 2.0 | | 86.0 / 14.0 / 0.0 | | AAAAAATTTAAATCTATAATT |
| 3 | 23,283 - 23,322 | | 14 | | 2.9 | | 85.0 / 15.0 / 0.0 | | GAAAAAAAAATATT |
| 4 | 23,918 - 23,959 | | 15 | | 3.0 | | 80.0 / 0.7 / 13.0 | | TAATTTAAATAATAT |

^a^Indices of the repeat in the mitochondrial genomic sequences.

^b^Size of the consensus repeat pattern.

^c^Number of copies that aligned with the consensus repeat pattern.

^d^Percent of Matches/Mismatches/Indels between adjacent copies of consensus repeat pattern.

**SUPPLEMENTARY TABLE S4 |** *Sordariomycetes* mitochondrial genomes that used for phylogenetic analyses in this study.

| Species name^a^ | Phylum (order : family) | Mitochondrial genome size (Kb) | GC  (%) | Number of gene  (total gene : protein CDS : rRNA : tRNA )^b^ | GenBank accession no. |
| --- | --- | --- | --- | --- | --- |
| *Acremonium chrysogenum* ATCC 11550 | Hypocreales : Hypocreales incertae sedis | 27.27 | 26.5 | 45 : 17 : 2 : 26 | KF757229.1 |
| *Acremonium fuci* 3a34 | Hypocreales : Hypocreales incertae sedis | 24.57 | 28.8 | 45 : 15 : 2 : 28 | KR864757.1 |
| *Akanthomyces muscarius* (*Lecanicillium muscarium*) | Hypocreales : Cordycipitaceae | 24.50 | 27.1 | 42 : 15 : 2 : 25 | AF487277.1 |
| *Beauveria bassiana* | Hypocreales : Cordycipitaceae | 29.96 | 27.2 | 42 : 15 : 2 : 25 | EU371503.2 |
| *Beauveria brongniartii* (*Cordyceps brongniartii* ) | Hypocreales : Cordycipitaceae | 33.93 | 27.3 | 42 : 20 : 2 : 25 | EU100743.1 |
| *Beauveria caledonica* | Hypocreales : Cordycipitaceae | 38.32 | 26.3 | 54 : 27 : 1 : 26 | KT201150.1 |
| *Beauveria malawiensis* | Hypocreales : Cordycipitaceae | 44.14 | 26.7 | 56 : 30 : 1 : 25 | KT201147.1 |
| *Beauveria pseudobassiana* C1010 | Hypocreales : Cordycipitaceae | 28.01 | 27.5 | 43 : 15 : 3 : 25 | KF297618.1 |
| *Clonostachys rosea* 6792 | Hypocreales : Bionectriaceae | 40.92 | 27.9 | 42 : 15 : 2 : 25 | KU668563.1 |
| *Cordyceps cicadae* CCAD02 | Hypocreales : Cordycipitaceae | 56.58 | 26.1 | 70 : 43 : 2 : 25 | MH922223.1 |
| *Cordyceps militaris* EFCC-C2 | Hypocreales : Cordycipitaceae | 33.28 | 26.8 | 43 : 15 : 2 : 26 | KF432176.1 |
| *Epichloe festucae* AR5 | Hypocreales : Clavicipitaceae | 88.75 | 27.5 | 85 : 60 : - : 25 | KX066186.1 |
| *Epichloe typhina* E8 | Hypocreales : Clavicipitaceae | 84.63 | 27.0 | 81 : 56 : - : 25 | KX066185.1 |
| *Fusarium circinatum* MRC 7870 | Hypocreales : Nectriaceae | 67.11 | 31.4 | 44 : 30 : 2 : 27 | JX910419.1 |
| *Fusarium commune* JCM11502 | Hypocreales : Nectriaceae | 47.53 | 32.4 | 46 : 18 : 2 : 26 | NC_036106.1 |
| *Fusarium culmorum* CBS 139512 | Hypocreales : Nectriaceae | 103.85 | 31.7 | 90 : 60 : 2 : 28 | KP827647.1 |
| *Fusarium gerlachii* CBS 123666 | Hypocreales : Nectriaceae | 93.43 | 31.9 | 83 : 53 : 2 : 28 | KM486533.1 |
| *Fusarium mangiferae* | Hypocreales : Nectriaceae | 30.63 | 31.3 | 28 : 18 : 1 : 9 | KP742838.1 |
| *Fusarium oxysporum* F11 | Hypocreales : Nectriaceae | 34.48 | 31.0 | 17 : 16 : 2 : 25 | AY945289.1 |
| *Fusarium verticillioides*  (*Gibberella moniliformis* ) 7600 | Hypocreales : Nectriaceae | 53.75 | 32.6 | 50 : 21 : 2 : 27 | NC_016687.1 |
| *Hirsutella minnesotensis* 3608 | Hypocreales : Ophiocordycipitaceae | 52.25 | 28.4 | 56 : 30 : 2 : 25 | KR139916.1 |
| *Hirsutella rhossiliensis* | Hypocreales : Ophiocordycipitaceae | 62.48 | 28.2 | 47 : 24 : 2 : 26 | KU203675.1 |
| *Hirsutella thompsonii* ARSEF 9457 | Hypocreales : Ophiocordycipitaceae | 62.51 | 29.8 | 59 : 30 : 2 : 27 | MH367294.1 |
| *Hirsutella vermicola* | Hypocreales : Ophiocordycipitaceae | 53.79 | 25.3 | 54 : 27 : 2 : 25 | KY465721.1 |
| *Ilyonectria destructans* | Hypocreales : Nectriaceae | 42.90 | 28.2 | 46 : 15 : 2 : 29 | KU881725.1 |
| *Lecanicillium saksenae* | Hypocreales : Cordycipitaceae | 25.92 | 26.5 | 43 : 15 : 2 : 26 | KT585676.1 |
| *Metarhizium anisopliae* ME1 | Hypocreales : Clavicipitaceae | 24.67 | 28.4 | 41 : 15 : 2 : 24 | AY884128.1 |
| *Nectria cinnabarina* 5175 | Hypocreales : Nectriaceae | 69.90 | 28.7 | 42 : 15 : 2 : 25 | KT731105.1 |
| *Ophiocordyceps sinensis* | Hypocreales : Ophiocordycipitaceae | 157.54 | 30.2 | 117 : 88 : 2 : 27 | KY622006.1 |
| *Parengyodontium album* | Hypocreales : Cordycipitaceae | 28.08 | 25.9 | 43 : 17 : 2 : 24 | KX061492.1 |
| *Pochonia chlamydosporia*  (*Metacordyceps chlamydosporia*) 170 | Hypocreales : Clavicipitaceae | 25.62 | 28.3 | 39 : 15 : 2 : 22 | KF479445.1 |
| *Tolypocladium inflatum ARSEF* 3280 | Hypocreales : Ophiocordycipitaceae | 25.33 | 27.8 | 42 : 15 : 2 : 25 | KY924879.1 |
| *Tolypocladium ophioglossoides* | Hypocreales : Ophiocordycipitaceae | 35.16 | 27.5 | 44 : 19 : 2 : 25 | KX455872.1 |
| *Trichoderma asperellum* B05 | Hypocreales : Hypocreaceae | 30.00 | 27.8 | 43 : 17 : 1 : 25 (1) | NC_037075.1 |
| *Trichoderma gamsii* KUC1747 | Hypocreales : Hypocreaceae | 29.30 | 28.3 | 47 : 18 : 2 : 26 (1) | KU687109.1 |
| *Trichoderma hamatum* | Hypocreales : Hypocreaceae | 32.76 | 27.7 | 48 : 20 : 2 : 26 (1) | MF287973.1 |
| *Trichoderma reesei* QM9414 | Hypocreales : Hypocreaceae | 42.13 | 27.2 | 44 : 19 : 2 : 24 (2) | AF447590.1 |
| *Fusarium solani* mpVI | Hypocreales : Nectriaceae | 62.98 | 28.9 | 57 : 30 : 2 : 25 | JN041209.1 |
| *Neurospora crassa* OR74A^c^ | Sordariales : Sordariaceae | 64.84 | 36.1 | 58 : 28 : - : 28 | KC683708.1 |
| *Trichoderma atroviride* ATCC 26799 | Hypocreales : Hypocreaceae | 32.76 | 28.2 | 51 : 21 : 2 : 27 (1) | MN125601 (this study) |

^a^All descriptions of mitochondrial genomic features of *Sordariomycetes* species were based on NCBI databases (datasets as of, December 2019).

^b^Non-existence of rRNAs were indicated with a dash (-) in the column; To the *Trichoderma* mitochondrial genomes, a number of putative *trn* genes predicted within the protein-coding genes were indicated inside the parentheses.

^c^Used as an outgroup for phylogenetic analyses in this study.

**SUPPLEMENTARY TABLE S5 |** Gene components that used for the comparative analysis of mitochondrial gene orders among *Trichoderma* species.

| Species name  Gene (product) | *T. atroviride*  ATCC 26799 | *T. reesei*  QM9414^a^ | *T. asperellum*  B05^a^ | *T. hamatum*^a^ | *T. gamsii*  KUC1747^a^ |
| --- | --- | --- | --- | --- | --- |
|  | Gene placement  (Start position – Stop position, Length (nt))^b^ | | | | |
| *atp6* (ATP synthase F0 subunit 6) | 14,161 - 14,943  (783) | 3,601 - 4,380  (780) | 24,539 - 25,321  (783) | 2,377 - 3,159  (783) | 7,876 - 8,658  (783) |
| *atp8* (ATP synthase F0 subunit 8) | 13,763 - 13,909  (147) | 3,287 - 3,439  (153) | 24,213 - 24,359  (147) | 2,043 - 2,189  (147) | 7,478 - 7,624  (147) |
| *atp9* (ATP synthase F0 subunit 9) | 31,610 - 31,834  (225) | 18,927 - 19,130  (204) | 10,876 - 11,100  (225) | 19,266 - 19,490  (225) | - |
| *cob* (apocytochrome b) | 3,498 - 6,182  (2,685) | 27,380 - 30,933  (3,554) | 17,024 - 18,193  (1,170) | 26,342 - 27,511  (1,170) | 1 - 1,170  (1,170) |
| *cox1* (cytochrome c oxidase subunit 1) | 6,801 - 9,672  (2,872) | 31,523 - 40,232  (8,710) | 18,848 - 20,434  (1,587) | 28,132 - 30,995  (2,864) | 1,790 - 3,382  (1,593) |
| *cox2* (cytochrome c oxidase subunit 2) | 32,009 - 32,758  (750) | 21,830 - 23,704  (1,875) | 11,269 - 12,018  (750) | 19,665 - 22,856  (3,192) | 25,061 - 25,810  (750) |
| *cox3* (cytochrome c oxidase subunit 3) | 17,536 - 18,345  (810) | 6,815 - 7,624  (810) | 27,675 - 29,602  (1,928) | 5,837 - 7,764  (1,928) | 11,878 - 12,078  (201) |
| *nad1* (NADH dehydrogenase subunit 1) | 10,326 - 11,435  (1,110) | 40,831 - 41,973  (1,143) | 21,062 - 22,171  (1,110) | 31,638 - 32,747  (1,110) | 3,993 - 5,150  (1,158) |
| *nad2* (NADH dehydrogenase subunit 2) | 29,342 - 31,009  (1,668) | 16,531 - 18,315  (1,785) | 8,600 - 10,267  (1,668) | 16,990 - 18,657  (1,668) | 22,394 - 24,061  (1,668) |
| *nad3* (NADH dehydrogenase subunit 3) | 31,010 - 31,423  (414) | 18,316 - 18,729  (414) | 10,268 - 10,681  (414) | 18,658 - 19,071  (414) | 24,062 - 24,475  (414) |
| *nad4* (NADH dehydrogenase subunit 4) | 11,647 - 13,104  (1,458) | 1 - 1,470  (1,470) | 22,313 - 23,770  (1,458) | 171 - 1,628  (1,458) | 5,362 - 6,819  (1,458) |
| *nad4L* (NADH dehydrogenase subunit 4L) | 292 – 561  (270) | 24,021 - 24,320  (300) | 12,329 - 13,618  (1,290) | 23,141 - 23,410  (270) | 26,099 - 26,368  (270) |
| *nad5* (NADH dehydrogenase subunit 5) | 561 - 2,627  (2,067) | 24,320 - 26,398  (2,079) | 14,075 - 16,135  (2,061) | 23,410 - 25,470  (2,061) | 26,368 - 28,434  (2,067) |
| *nad6* (NADH dehydrogenase subunit 6) | 18,638 - 19,390  (753) | 7,964 - 8,734  (771) | 192 – 698  (507) | 8,058 - 8,810  (753) | 12,378 - 13,130  (753) |
| *rns* (small subunit ribosomal RNA) | 15,514 - 17,017  (1,504) | 4,858 - 6,247  (1,390) | 25,676 - 27,177  (1,502) | 3,753 - 5,241  (1,489) | 9,249 - 10,750  (1,502) |

^a^Based on the GenBank database on NCBI (datasets as of, December 2019).

^b^Non-existence of target genes in the mitochondrial genome was indicated with a dash (-) in the column.

**SUPPLEMENTARY TABLE S6 |** Obtained values of Ka/Ks ratios for 13 core protein-coding genes in the mitochondrial genome of *T. atroviride* ATCC 26799.

| gene pairs between^a^ | Ka (nonsynonymous substitution rate) / Ks (synonymous substitution rate) ratio^b^ | | | | | | | | | | | | |
| --- | --- | --- | --- | --- | --- | --- | --- | --- | --- | --- | --- | --- | --- |
|  | *atp6* | *atp8* | *cob* | *cox1* | *cox2* | *cox3* | *nad1* | *nad2* | *nad3* | *nad4* | *nad4L* | *nnad5* | *nad6* |
| TS-T1 | 0.016 | 0.144 | 0.129 | 0.105 | 0.221 | 0.114 | 0.033 | 0.100 | 0.127 | 0.135 | 0.000 | 0.017 | 0.072 |
| TS-T2 | 0.026 | 0.000 | 0.063 | 0.023 | 0.067 | 0.000 | 0.022 | 0.143 | 0.150 | 0.145 | 0.774 | 0.026 | 0.043 |
| TS-T3 | 0.016 | 0.000 | 0.068 | 0.062 | 0.067 | 0.000 | 0.020 | 0.143 | 0.290 | 0.190 | 0.000 | 0.026 | 0.096 |
| TS-T4 | 0.000 | 0.000 | 0.127 | 0.221 | 0.000 | 0.282 | 0.000 | 0.233 | 0.000 | 0.000 | 0.000 | 0.000 | 0.000 |

^a^TS, *T. atroviride* ATCC 26799 (GenBank accession no. MN125601, in this study); T1, *T. reesei* QM9414 (GenBank accession no. AF447590.1); T2, *T. asperellum* B05 (GenBank accession no. NC_037075.1); T3, *T. hamatum* (GenBank accession no. MF287973.1); T4, *T. gamsii* KUC1747 (GenBank accession no. KU687109.1).

^b^Based on the pairwise alignments of target genes (one-to-one gene pairs) among *Trichoderma* species

**SUPPLEMENTARY TABLE S7 |** Predicted putative tRNA gene (*trn* gene) within the protein-coding gene of *Trichoderma* mitochondrial genomes.

| Species  (GenBank assession no.) | Total number of putative *trn* gene | Replacement  (predicted putative tRNA : Length (nt) : overlap position) |
| --- | --- | --- |
| *T. atroviride*  ATCC 26799  (MN125601, this study) | 1 | tRNA^Val^ : 72 bp (19,308 - 19,379)^b^ : overlapped with the 3’ end of the *nad6* gene (18,638 - 19,390)^c^ |
| *T. reesei* QM9414 (AF447590)^a^ | 2 | tRNA^Val^ : 72 bp (8,652 - 8,723)^b^ : overlapped with the 3’ end of the *nad6* gene (7,964 - 8,734)^c^  tRNA^Met^ : 72 bp (16,538 - 16,609)^b^ : overlapped with the 5’ end of the *nad2* gene (16,531 - 18,315)^c^ |
| *T. asperellum* B05 (NC_037075)^a^ | 1 | tRNA^Val^ : 72 bp (616 - 687)^b^ : overlapped with the 3’ end the *nad6* gene (192 – 698)^c^ |
| *T. hamatum*  (MF287973)^a^ | 1 | tRNA^Val^ : 72 bp (8,728 - 8,799)^b^ : overlapped with the 3’ end of the *nad6* gene (8,058 - 8,810)^c^ |
| *T. gamsii* KUC1747 (KU687109)^a^ | 1 | tRNA^Val^ : 72 bp (13,048 - 13,119)^b^ : overlapped with the 3’ end of the *nad6* gene (12,378 - 13,130)^c^ |

^a^Based on the GenBank database on NCBI (datasets as of, December 2019).

^b^Start position - Stop position of a putative tRNA gene in the mitochondrial genome.

^c^Start position - Stop position of each protein-coding gene in the mitochondrial genome.
