# Supplementary figures and images for: Complete Mitochondrial Genome of the Fungal Biocontrol Agent Trichoderma atroviride: Genomic Features, Comparative Analysis and Insight Into the Mitochondrial Evolution in Trichoderma
Source: Front Microbiol. 2020 Apr 28;11:785. doi: 10.3389/fmicb.2020.00785 (PMC7228111; doi:10.3389/fmicb.2020.00785)

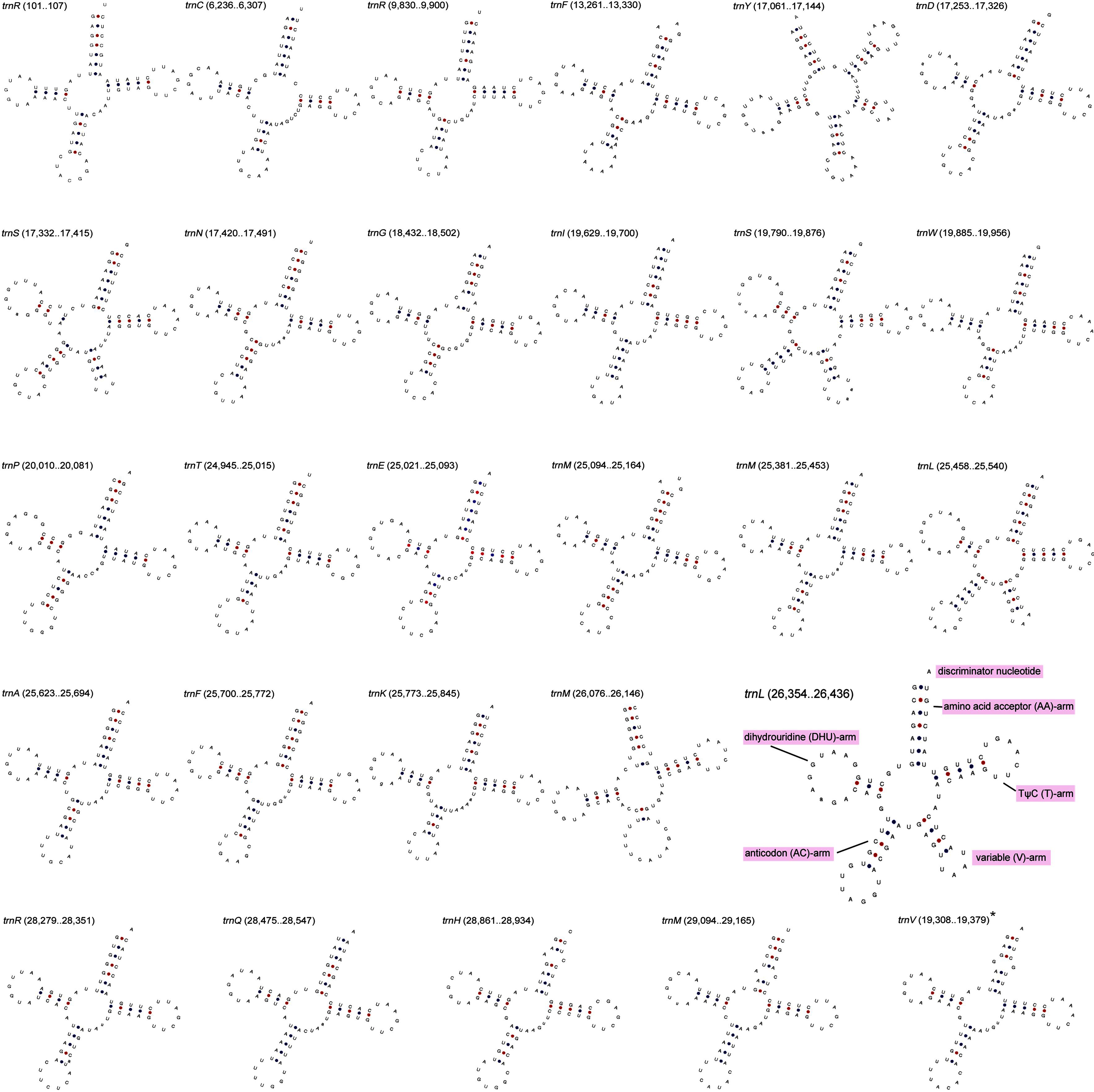

Supplement: Supplementary file 3 [file Image_1.JPEG]

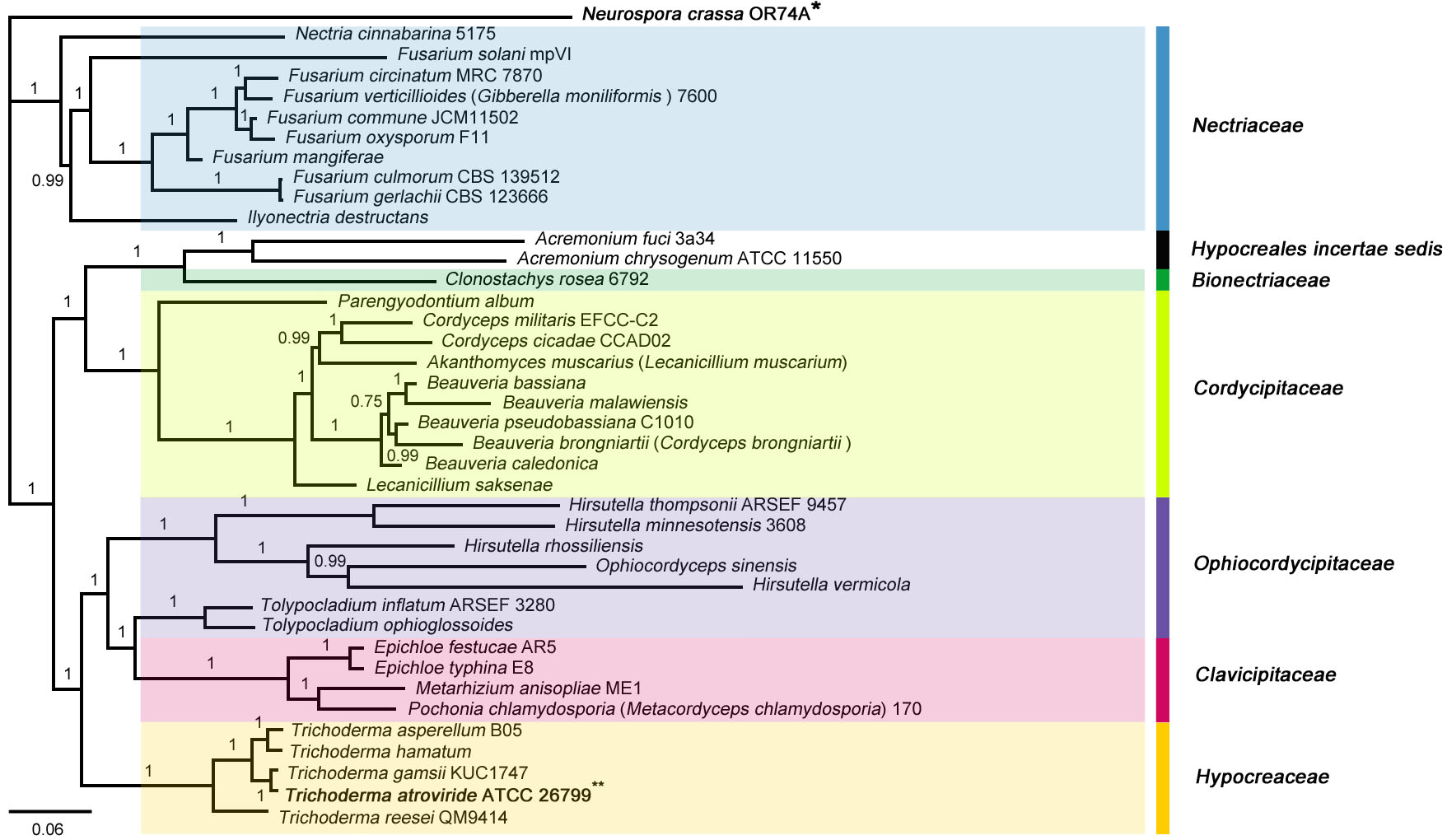

Supplement: Supplementary file 4 [file Image_2.JPEG]

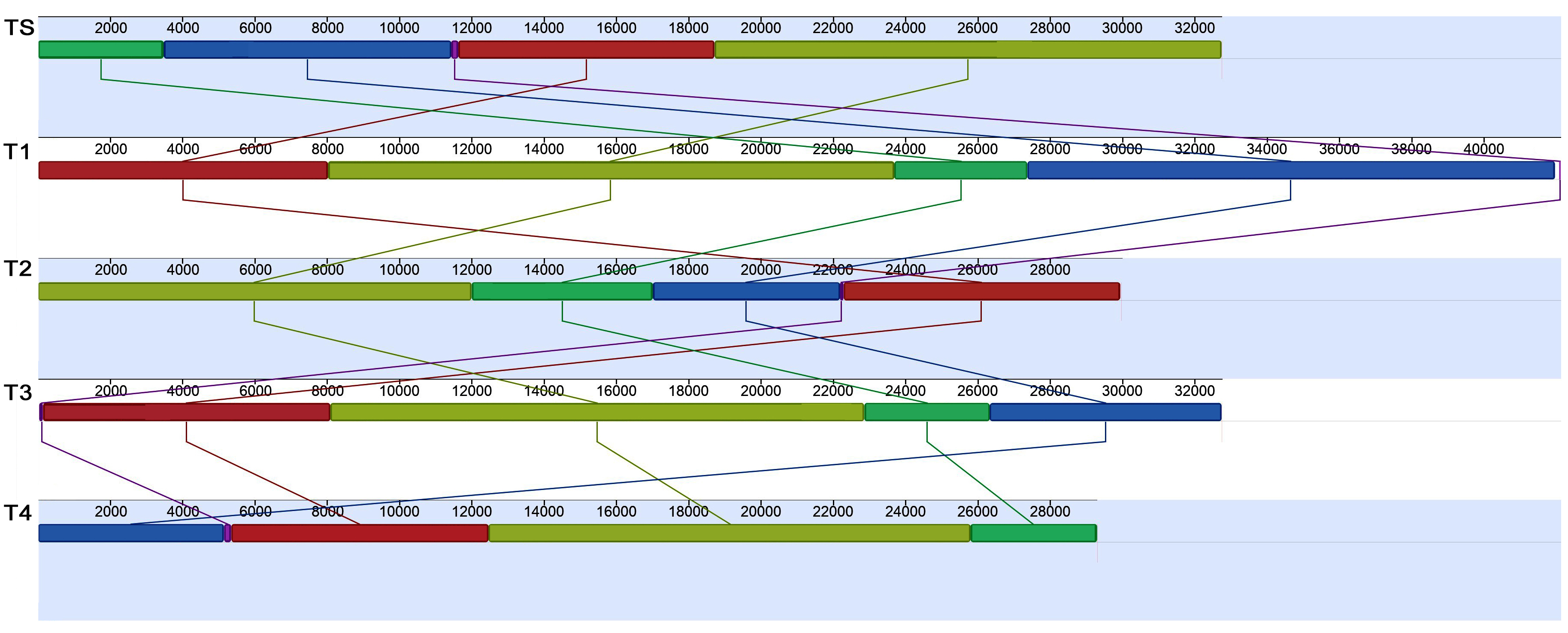

Supplement: Supplementary file 5 [file Image_3.JPEG]
